# Build Back Worse: The Media Coverage of Well-being Metrics Before and During the COVID-19 Pandemic in the Crucial Cases of Scotland and Italy

**DOI:** 10.1007/s11205-022-03037-x

**Published:** 2023-02-28

**Authors:** Fabio Battaglia

**Affiliations:** The London School of Economics and Political Science, London, UK

**Keywords:** Well-being, Beyond GDP, Media coverage, Build Back Better, COVID-19

## Abstract

Despite the media are often described as critical for the success of the well-being agenda, there is wide dissatisfaction with their current level of interest. However, the media coverage of well-being metrics has been unresearched and, even when studies have been conducted, these employed unrobust methodologies, were limited to newspapers and to restricted samples of metrics. This paper fills such gap, providing also for the first time an analysis of radio and TV coverage of well-being metrics. The research was undertaken using Factiva (for newspapers) and TVEyes (for radio and TV) for the years of 2017–2021 and 2018–2021, respectively. The countries analysed are Scotland and Italy, both pioneers in the measurement of well-being. Findings reveal that media coverage of well-being metrics has been extremely low overall and that this was impacted negatively by the COVID-19 pandemic, which instead impacted positively on the reporting of GDP and related queries, showing that the main concern during the pandemic was the impact that this was going to have in terms of output, rather than in terms of well-being. Most composite indices, whose creation is often thought to help obtain greater media coverage, were almost if not even fully ignored by journalists, whereas metrics that lack an overall composite index but that are overseen by independent institutions and have been institutionalised were among the ones that were reported the most.

## Introduction

While different indicators exist that could be used to measure well-being, Gross Domestic Product (GDP), the measure of a country’s economic output, is frequently said to have become the predominant one. According to the European Commission (2009, p. 2), it has ‘come to be regarded as a proxy indicator for overall societal development and progress’ whereas for Stiglitz, Sen and Fitoussi (2009, p. 7) it is now used to assess ‘everything – performance, well-being, quality of life’. Such use of GDP is problematic for many reasons for GDP, among other things, excludes activities that contribute to well-being such as volunteering and any type of unpaid work (Colman, 2021; Diener & Seligman, 2004), it does not take into account the depletion of natural resources (Büchs & Koch, 2017; Colman, 2021; Stiglitz et al., 2009) and it tells us nothing about income inequality (Colman, 2021; Laurent, 2018; Stiglitz et al., 2009). This has sparked a debate worldwide about the importance of measuring well-being adequately and more holistically (e.g. Costanza et al*.*, 2009, 2013, 2014; Fioramonti, 2013, 2014; Stiglitz et al., 2009), which has led to the development of a vast array of well-being metrics (see Bandura, 2008; Yang, 2014).

The success of these metrics was claimed in many studies to depend on their level of media coverage. For POINT (Policy Influence of Indicators) workshop participants, for instance, success for well-being metrics meant primarily to be ‘quoted, mentioned and reported on an ongoing basis’ (Bell & Morse, 2013, p. 21). Journalists’ support was also the very first thing CONCORD (Confederation of European NGOs for Relief and Development) seminar participants highlighted (CONCORD, 2017), and the role of the media was defined as ‘critical’ by Wallace and Schmuecker (2012, p. 42). Similarly, according to BRAINPOoL (Bringing Alternative Indicators Into Policymaking) researchers, ‘[f]or Beyond GDP indicators there is an imperative to be attractive to the media: No media coverage equals no pressure on politicians and influence in politics’ (Hák et al*.*, 2012, p. 14). Media coverage is therefore one among several indicators that can be used to gauge the success of the well-being agenda. In fact, since journalists often report what policymakers say, this also tells us whether and how frequently policymakers have been referring to well-being metrics publicly, which in turn reveals what is or what is not on the political agenda and what metrics are debated when decisions are being taken. Media coverage can thus reveal the salience of metrics, and, albeit limitedly and imperfectly, be also an indirect proxy for policy impact (see Hák et al., 2012). Nevertheless, despite the crucial role that the media are thought to play, there is wide dissatisfaction with the coverage that well-being metrics have received so far. The European Commission (2013, p. 17) lamented that they ‘are rarely commented on in the media’ – with the perceived lack of media support highlighted as a barrier to the success of the well-being agenda in several countries (e.g. Chancel, Thiry and Demailly, 2014; Wallace & Schmuecker, 2012) –, whereas Fioramonti (2013, p. 115) argued that ‘the media hunger is all focused on GDP’.

At the forefront of the well-being debate are the crucial cases (Eckstein, 2011) of Scotland and Italy, both often regarded as model examples or best practices (e.g. Boarini & Smith, 2014; Durand, 2018; Exton & Shinwell, 2018; Noll, 2014). Scotland, a success story according to Stiglitz (OECD, 2012), showed interest in the well-being agenda before many other countries, with its official well-being framework, the National Performance Framework (NPF), launched by the Scottish Government as early as 2007. This has since been refreshed several times, the last one in 2018 when the Scottish Government also co-established, together with the Governments of Iceland and New Zealand, the Wellbeing Economy Governments network. Italy has a much longer and established history of well-being measurement, and several metrics were launched in the 1990s already. It was however in the late 2000s, with the appointment as President of the Italian National Institute of Statistics (ISTAT) of Enrico Giovannini that works began for the development of an official well-being framework, with the first Equitable and Sustainable Well-being (BES, according to the Italian acronym) Report published in 2013 after a public consultation which included the surveying of a representative sample of 54,000 people (Giovannini & Rondinella, 2012). In 2016, well-being indicators were embedded into national legislation and the Ministry of the Economy and Finance (MEF) was mandated to issue two documents annually: an Appendix to the Economic Planning Document (DEF) – the country’s budgetary plan – which monitor the trend of the selected indicators over the previous three years and make forecasts about the next three ones, and a Report on their trends to be presented to parliament.

Surprisingly, despite the key role that the media are thought to play the media coverage of well-being metrics remains an unexplored field, especially in crucial countries like Scotland and Italy which are at the forefront of the well-being debate. This article provides the first ever analysis of media coverage of well-being metrics in both countries and is structured as follows. First, I review the evidence currently available on the topic, discussing its limitations. After explaining the methodology that I employed, I show and discuss the findings of the research before offering some final remarks and recommendations.

## Evidence Available on the Media Coverage of Well-being Metrics

While claims regarding the low media uptake of well-being metrics remain largely unsubstantiated, there is some evidence suggesting that the coverage of well-being metrics has been limited to date. As part of POINT, Morse (2011a, b, 2013) investigated the coverage received between 1990 and 2009 in the United Kingdom (UK) by three indices (the Human Development Index or HDI, the Ecological Footprint and the Corruption Perception Index) in several newspapers (33, 22 and 31 for each study, respectively) using the NewsUK database. Morse found several articles mentioning the above indices (2011a) with peaks of reporting in the months in which metrics were issued (2011b, 2013). Nevertheless, Morse (2013, p. 247) concluded that this could ‘hardly be called an extensive coverage’ since the overall number of articles was less than 2.5% compared to the number of articles that mentioned GDP at least once over the same period.

However, the methodology employed by Morse was inconsistent, not transparent and incomplete. First, in two of his studies (2011b, 2013) he included among British newspapers the *Irish Times* because ‘widely available through the UK’ (2011a, p. 23). Yet, that contradicted Morse’s aim to study the UK press, not to mention that references to metrics found in articles belonging to the Irish press will have most likely referred to the ranking of Ireland. Since the *Irish Times* accounted for 15% of all articles (more than Scottish and Northern Irish newspapers combined), its inclusion clearly distorted his findings. Second, Morse (2011a, p. 1682) justified the selection of the three indices on the grounds that they had ‘been around for some years’ and had a ‘powerful backer’. This begs the question of why he excluded indices with the same characteristics such as the Living Planet Index (WWF LPI). Third, Morse did not specify why he changed the number of newspapers he searched every time and he only searched for indices by their full names, ignoring their acronyms, the name of the publications the indices were published in (e.g. *Human Development Report*) or in fact any other alternative names (e.g. Index of Human Development). Therefore, his figures largely underestimate the number of articles published that cited the indices in question.

Morse (2015, 2016, 2018) re-conducted similar studies after POINT. This time, he focused on the global press and on a higher number of metrics, using however a different database, NexisLexis. The findings of the three studies look very similar, although we are never told the overall number of articles yielded, or that for each metric, or in what countries articles were published. In fact, the methodology Morse employed was again rich in flaws. First, in his 2015 study he selected metrics that had been updated at least 10 times, whereas in his 2016 one he decreased that to 6, without explaining why.^1^ Second, Morse only focused on composite indices, again with little explanation. Third, despite focusing on the global press, Morse only searched for keywords in English (which, by the way, he never provided) based on debatable assumptions,^2^ meaning his findings hugely underestimate the actual number of relevant articles published worldwide. Fourth and most importantly, Morse did not consider that the coverage start date of sources included in NexisLexis varies from source to source. *The Guardian*, for instance, is covered from July 14, 1984, whereas the Italian *Corriere della Sera* from January 27, 2009, although both were founded much earlier. This means that during the period he analysed (which is unclear^3^) the former will have yielded more articles than the latter, though the latter might have published more. This also means that surges of articles in certain years were not due to a sudden increase in interest but simply to sources being added to the database.

As part of IN-STREAM (Integrating Mainstream Economic Indicators with Sustainable Development Objectives), a similar study was undertaken to investigate the media coverage of 19 metrics in several English and French newspapers from 1990 to 2010. Although the overall number of mentions and that for each metric were not shown, the study found the Ecological Footprint to be the most mentioned one, followed by the HDI (Bassi, et al., 2011). However, the methodology employed was scarcely explained and flawed. First, researchers were interested in newspapers, yet 4 out of 14 sources were news agencies. Second, it was not explained how metrics were searched for and in what language. Third, the coverage start date issue mentioned above was again overlooked.

BRAINPOoL conducted a similar study, finding 589,660 documents on ProQuest mentioning GDP and 25,628 mentioning the 15 metrics that the project focused on (Hák et al., 2012). However, the methodology employed was once again rich in flaws. First, the GPI was misspelled as Genuine Progress Index (the precursor of the Canadian Index of Wellbeing) instead of Genuine Progress Indicator. Given that ProQuest includes Canadian newspapers, this made researchers count irrelevant articles and reach the wrong conclusions, especially as they considered that of the GPI ‘successful in terms of […] media coverage’ (2012, p. 38). Second, ProQuest includes theses and academic journals among other things, excluding outlets such as radio and television (TV). Yet, the authors presented their results in terms of ‘media coverage’ (2012, p. 39). Third, queries were only searched for in English and it is not clear whether acronyms were used. Fourth and last, the coverage start date issue mentioned above was also in this case overlooked.

## Methodology

Despite the key role that the media have been said to play, the media coverage of well-being metrics has been insufficiently researched and, even when studies have been conducted, these employed unrobust methodologies, were limited to newspapers (or contrarily extended to all sorts of sources) and to restricted samples of metrics. A rigorous analysis of media coverage of well-being metrics is therefore lacking. By using Factiva for the analysis of newspaper coverage and TVEyes for that of radio and TV, I was able to fill this research gap. Factiva enables us to search over 45,000 sources (including, but not limited to, newspapers). TVEyes is a media monitoring tool that uses speech recognition technology to provide real-time transcripts of whatever is broadcast on radio and TV.^4^ Detailed information about both tools, including a thorough explanation of what and how sources were selected, what years were chosen and how duplicates were eliminated can be found in Appendix 1. A list of all sources covered can instead be found in Appendix 2 (for newspapers) and 3 (for radio and TV), whereas a detailed list of all strings and queries searched for can be found in Appendix 4 (for Scotland) and 5 (for Italy).

The list of metrics that I searched for can be found below (Table 1). All metrics were identified through a literature review of academic articles, policy documents, independent reports and newspaper articles produced about the well-being agenda in both countries conducted as part of a broader project on the use and impact of well-being metrics in Scotland and Italy. Such metrics are or used to be calculated for the country whose sample they belong to. In the case of Scotland, only the GIP, the HKI, the NPF and the Bank of Scotland QoLS are Scotland-specific. This does not mean that the remaining metrics are irrelevant as they do look at Scotland but in the wider context of the UK. Metrics that are or were not calculated for either country were excluded. The only exception is the GNH, which according to a journalist himself has ‘captured the media’s imagination’ (Speroni, 2010, p. 118), so much so that for another journalist Bhutan ‘has become symbolic of the happiness debate’ (Pilling, 2018, p. 263), probably because its name and acronym can be easily counterposed to GDP. Note that almost all metrics selected are composite indices, which makes the study even more interesting given that their creation is usually justified on the grounds that their simplicity will gain media traction (see Wallace & Schmuecker, 2012).

**Table 1:**
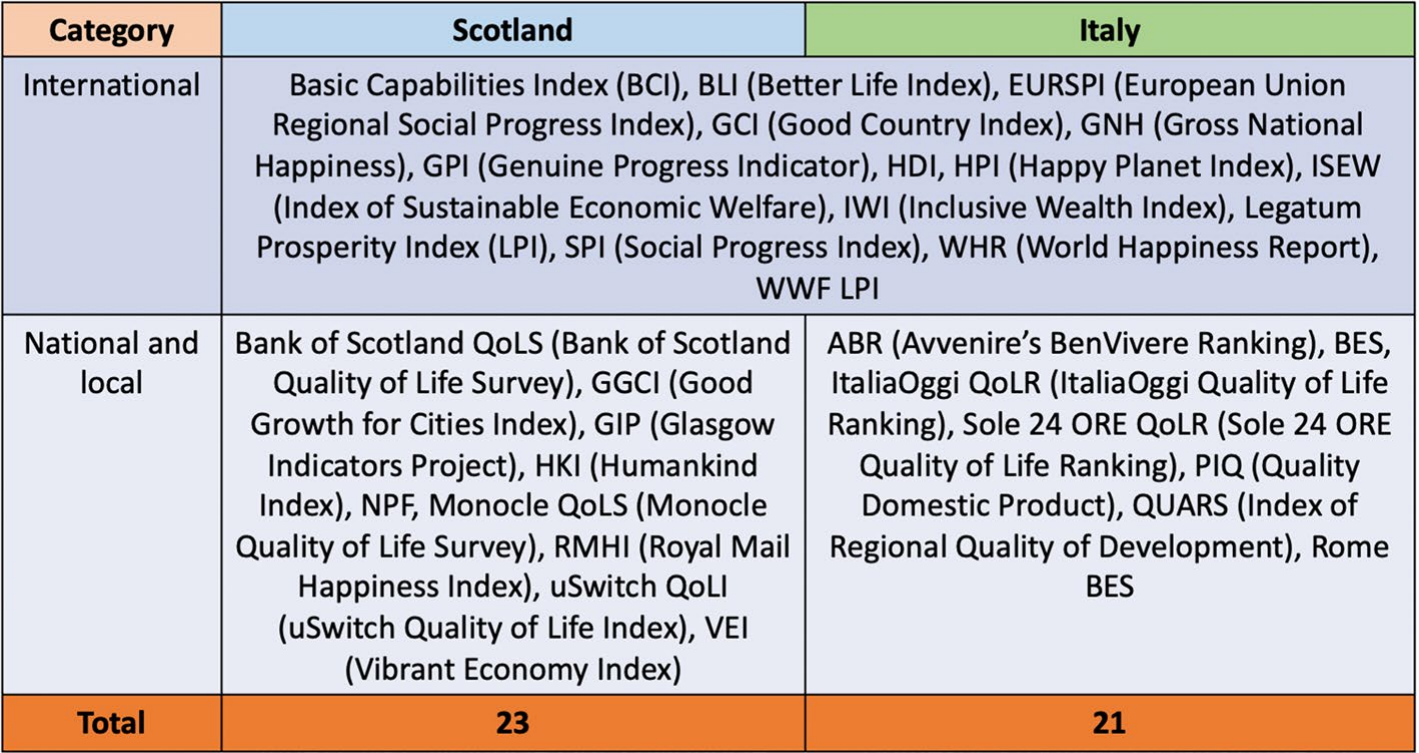
List of metrics searched for

## Results

### Newspaper Coverage – Scotland

**Fig. 1:**
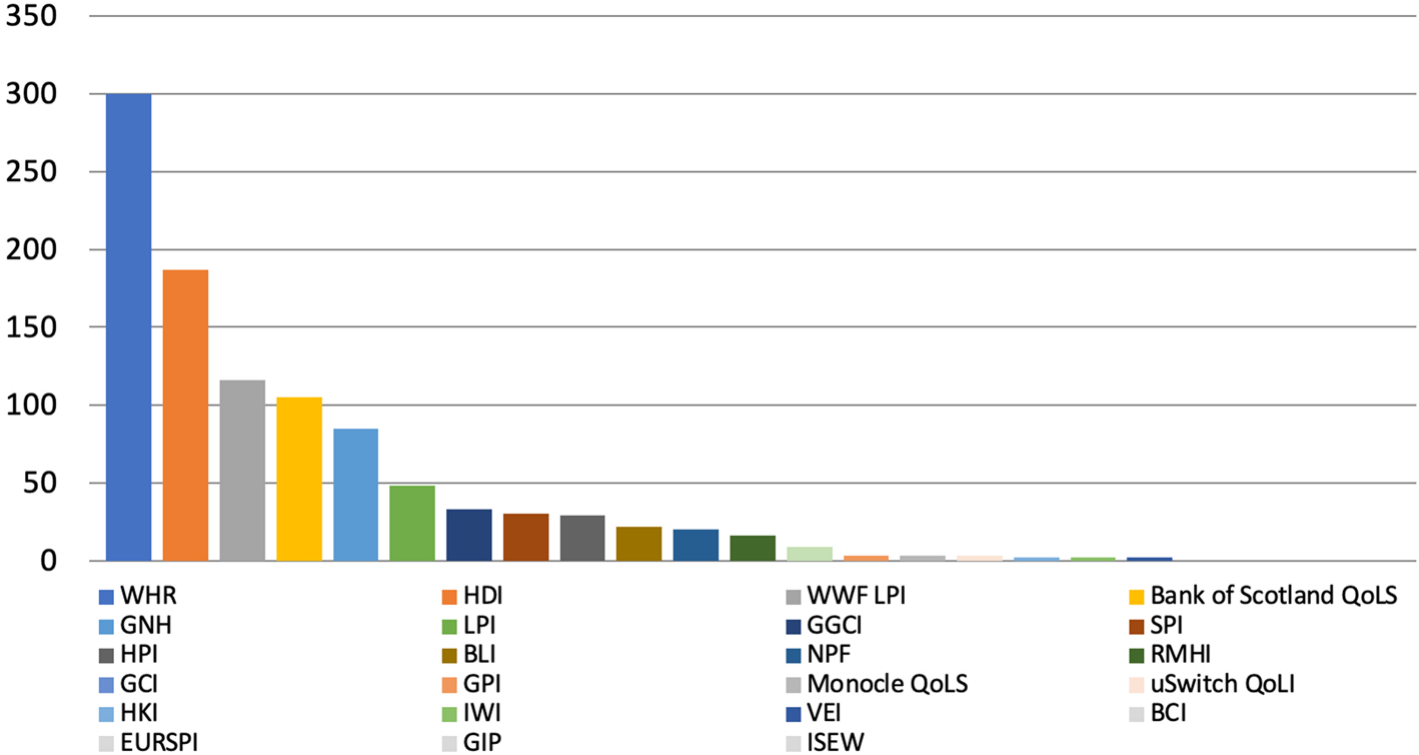
Number of articles published on 37 Scottish and UK‐wide newspapers mentioning at least once 23 well‐being metrics by metric, 2017–2021, duplicates excluded. Source: Factiva (author’s own elaboration)

The WHR is the metric that was found in the highest number of articles (300), followed by the HDI (187) and the WWF LPI (116) with the BCI, the EURSPI, the GIP and the ISEW at the end of the scale (all of which returned 0 results each), for a total of 1,015 articles (Fig. 1).

**Fig. 2:**
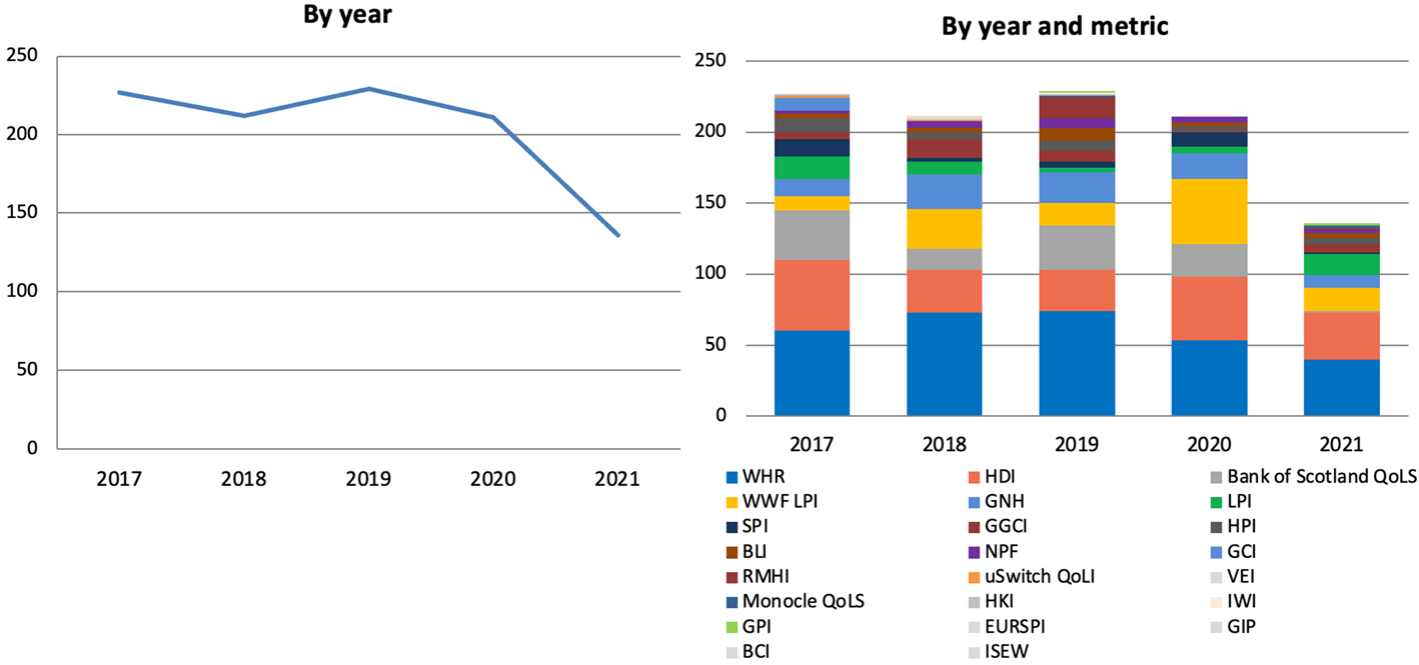
Number of articles published on 37 Scottish and UK-wide newspapers mentioning at least once 23 well-being metrics by year and by year and metric, 2017–2021, duplicates excluded. Source: Factiva (author's own elaboration)

The number of articles remained quite stable initially, only to start decreasing by 8% in 2020 and by 36% in 2021 (Fig. 2). Such initial decrease in 2020 may be due to the RMHI not being iterated and to the release of the 2020 GGCI being postponed to 2021. However, articles decreased despite the release of the WWF LPI which was not issued in 2019 being this a biannual publication and which was mentioned in more articles in 2020 (46) than the above metrics combined in 2019 (23). Similarly, the sharp decrease in 2021 may be due to the WWF LPI not being iterated then, but the overall gap in articles between 2020 and 2021 (75) is greater than the gap in articles published in the same years about the WWF LPI (30). Such overall decrease could then be due to the Bank of Scotland QoLS not being iterated in 2021, however 2021 saw the publication of the 2020 GGCI and the revival of the HPI, first updated since 2016. Therefore, such overall reduction in coverage, which can only limitedly be attributed to changes in the years metrics were released and which in fact occurred despite that, is most likely due to external factors, particularly the Coronavirus Disease 2019 (COVID-19) pandemic and the economic crisis that followed.^5^

**Fig. 3:**
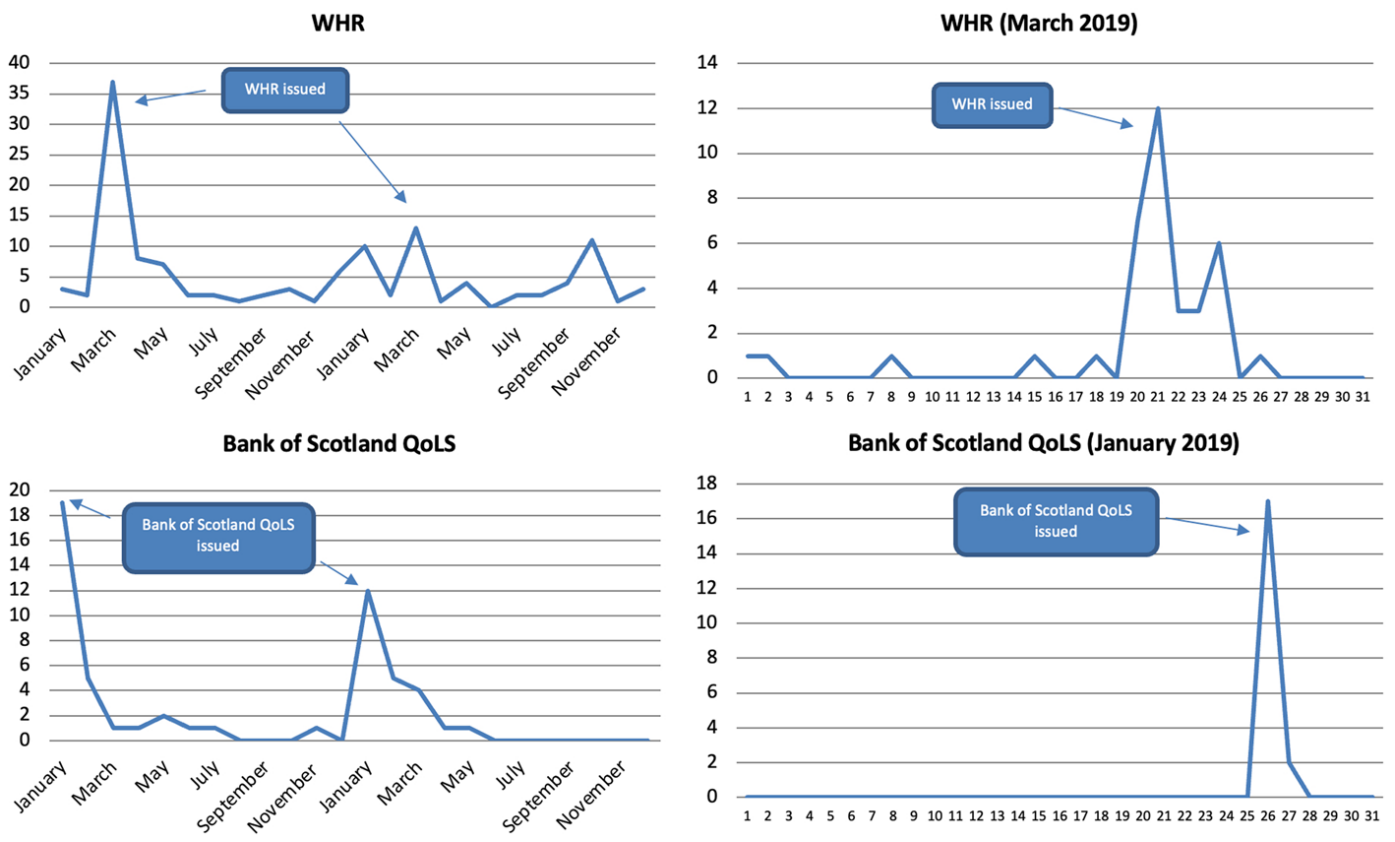
Number of articles published on 37 Scottish and UK-wide newspapers mentioning at least once selected well-being metrics by month (2019–2020) and by day (selected years and months), duplicates excluded. Source: Factiva (author's own elaboration)

The majority of articles were published within the months in which metrics were released (except for those that are not updated regularly such as the GNH). For instance, below are articles sorted by month for the WHR and the Bank of Scotland QoLS, which 40% of all articles refer to (Fig. 3). The major peaks were reached when the metrics in question were issued. In fact, articles were published almost exclusively on the day of their release.

**Fig. 4:**
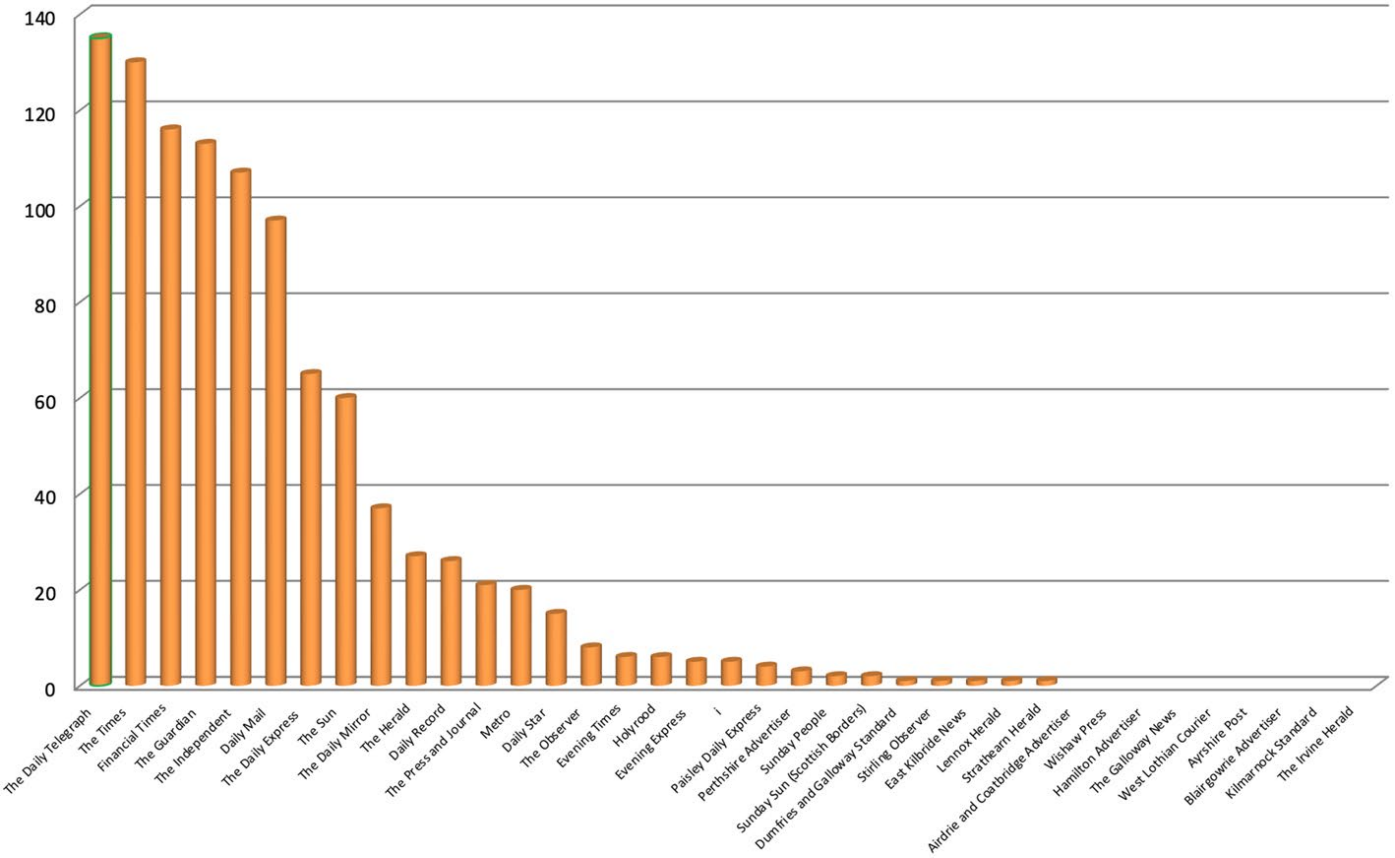
Number of articles published on 37 Scottish and UK-wide newspapers mentioning at least once 23 well-being metrics by source, 2017–2021, duplicates excluded. Source: Factiva (author’s own elaboration)

More than half (59%) of all articles were published in just five newspapers, namely *The Daily Telegraph, The Times, Financial Times, The Guardian* and *The Independent* (Fig. 4). No article was published in 9 newspapers and the last 14 sources that did publish at least one issued less than 10 each. Almost the entirety (90%) of all articles appeared in UK-wide sources. While some of these include Scotland editions, meaning some of the articles will include Scotland-specific content, regional and local newspapers were almost completely uninterested. On the one hand, this is due to the greater number of UK-wide metrics in the sample, which 87% of total articles refer to. On the other hand, UK-wide metrics do include Scotland and the number of UK-wide and Scottish newspapers in the sample is almost identical, which suggests that the well-being agenda has gained less traction in Scotland than it has in the UK overall. Furthermore, it is worth noting that *Holyrood*, a Scottish magazine dedicated exclusively to Scottish politics which often features interviews with Members of the Scottish Parliament and live updates from the First Minister’s Questions, only published a handful of articles, which suggests that well-being metrics were not part of Scottish policymakers’ language during the period analysed.

**Fig. 5:**
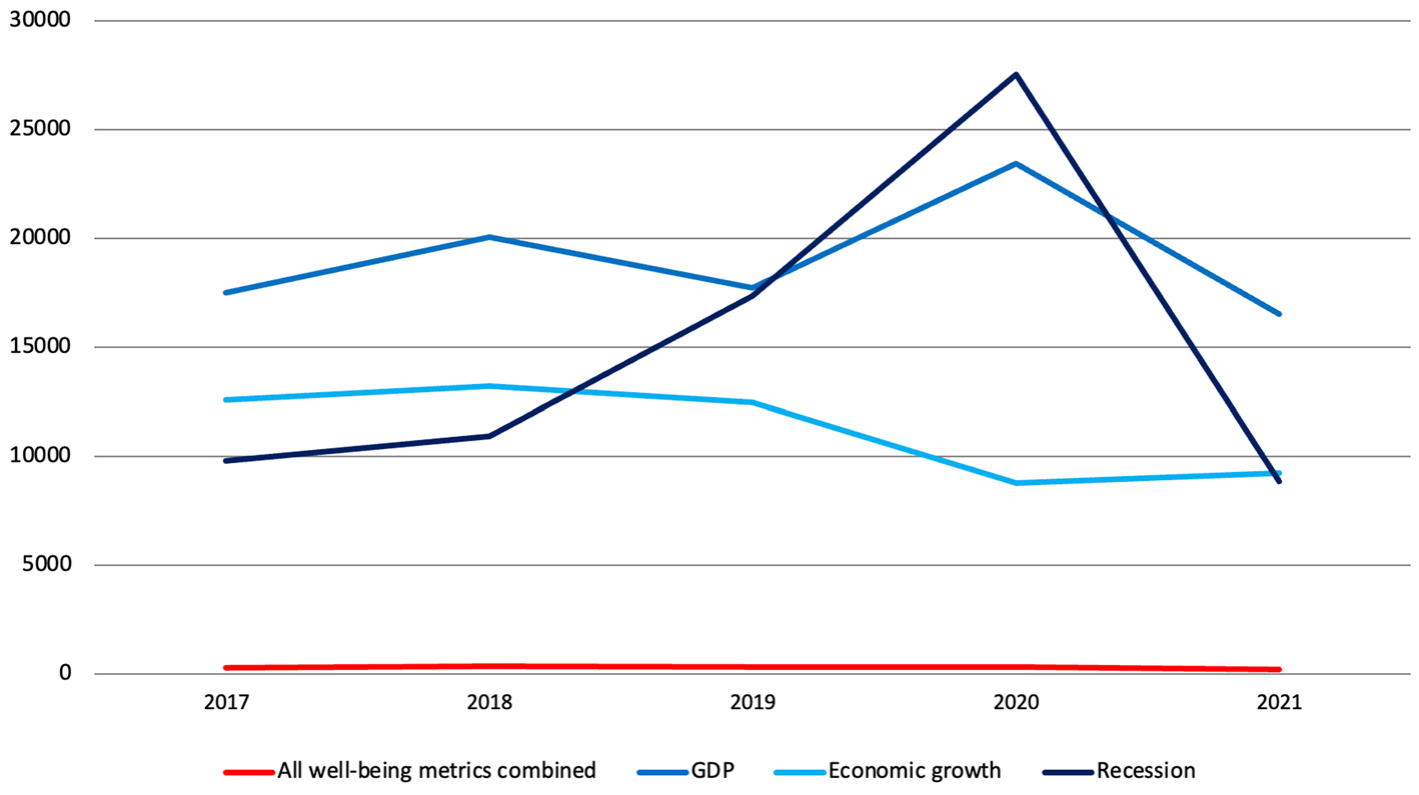
Number of articles published on 37 Scottish and UK-wide newspapers mentioning at least once 23 well-being metrics combined compared to GDP, economic growth and recession, by year, 2017–2021, duplicates included. Source: Factiva (author’s own elaboration)

The number of articles mentioning all 23 well-being metrics combined is 1.6% compared to the number of articles that mentioned at least once GDP over the same period, 2.7% compared to economic growth and 2% compared to recession (Fig. 5). Compared to 2019, the number of articles mentioning at least once either GDP or recession increased in 2020 by 32% and 59%, respectively, to which corresponded a 30% decrease in the number of articles relating to economic growth. The number of articles mentioning all well-being metrics combined once again decreased since 2019 reaching its lowest point in 2021, when articles covering GDP and recession went back to their 2018 levels and those relating to economic growth increased by 5%. The coverage of GDP and related terms thus seemed to follow the performance of the Scottish and UK economies during the COVID-19 pandemic, suggesting that of particular concern was the impact this was having and going to have primarily on GDP and in terms of economic output than in terms of and on well-being.

### Radio and TV Coverage – Scotland

**Fig. 6:**
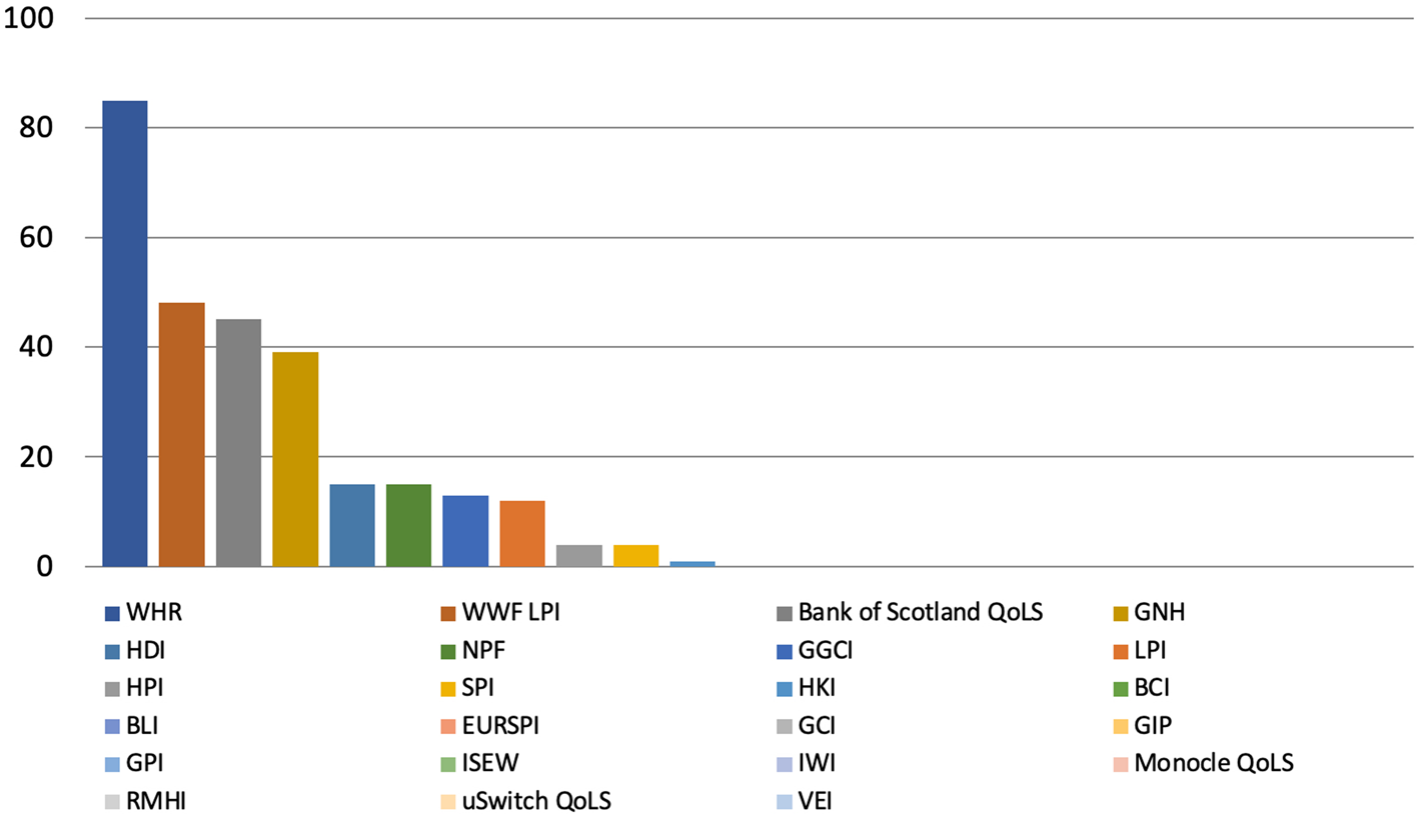
Number of references to 23 well-being metrics on 32 Scottish and UK-wide radio stations and 13 Scottish and UK-wide TV channels by metric, November 2018-December 2021, duplicates excluded. Source: TVEyes (author’s own elaboration)

The most mentioned metric over the 38-month period analysed was the WHR with 85 references, followed by the WWF LPI (48) and the Bank of Scotland QoLS (45), with 12 metrics at the end of the scale such as the BLI and the ISEW (all of which returned 0 results each), for a total of 281 mentions (Fig. 6).

**Fig. 7:**
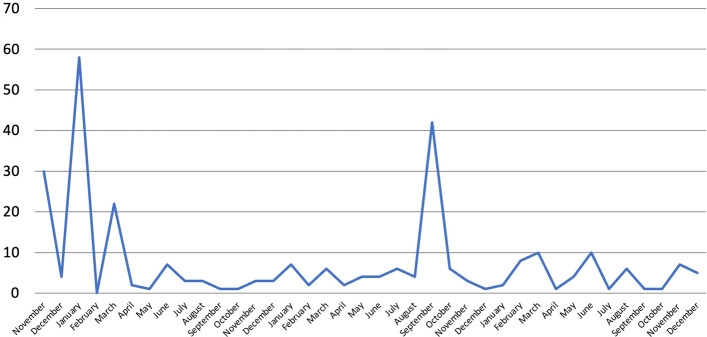
Number of references to 23 well-being metrics on 32 Scottish and UK-wide radio stations and 13 Scottish and UK-wide TV channels, by month, November 2018-December 2021, duplicates excluded. Source: TVEyes (author’s own elaboration)

Overall, the number of mentions decreased by 16% in 2020 compared to 2019 (Fig. 7). All metrics that were published in both years saw their number of mentions decrease (e.g. WHR, –28%; Bank of Scotland QoLS –100%), except for the SPI (+ 400%) whose increase becomes irrelevant when we consider that this went from 0 references in 2019 to 4 in 2020. Total mentions decreased despite the publication of the 2020 WWF LPI. In fact, if WWF LPI figures for the month of September 2020 when this was issued are excluded, the decrease is even larger: −54%. One possible explanation for this contraction could be that the 2020 GGCI was published in January 2021 and not in 2020. However, the GGCI was only reported 3 times in 2019, so this cannot have caused such a large decrease. Another explanation could be, in relation to the 2020 Bank of Scotland QoLS at least, that Orkney ranked best place to live for the eighth year in a row (i.e. there was no big story to report anymore). However, the year before this was reported despite Orkney ranking first for the seventh year in a row (i.e. there was no big story then either). It is thus highly plausible that the main reason behind this contraction was again the COVID-19 pandemic and the economic crisis that followed, whose effects kept showing throughout 2021 when overall references further decreased by 36% despite the revival of the HPI and the issuing of the GGCI (see above).

**Fig. 8:**
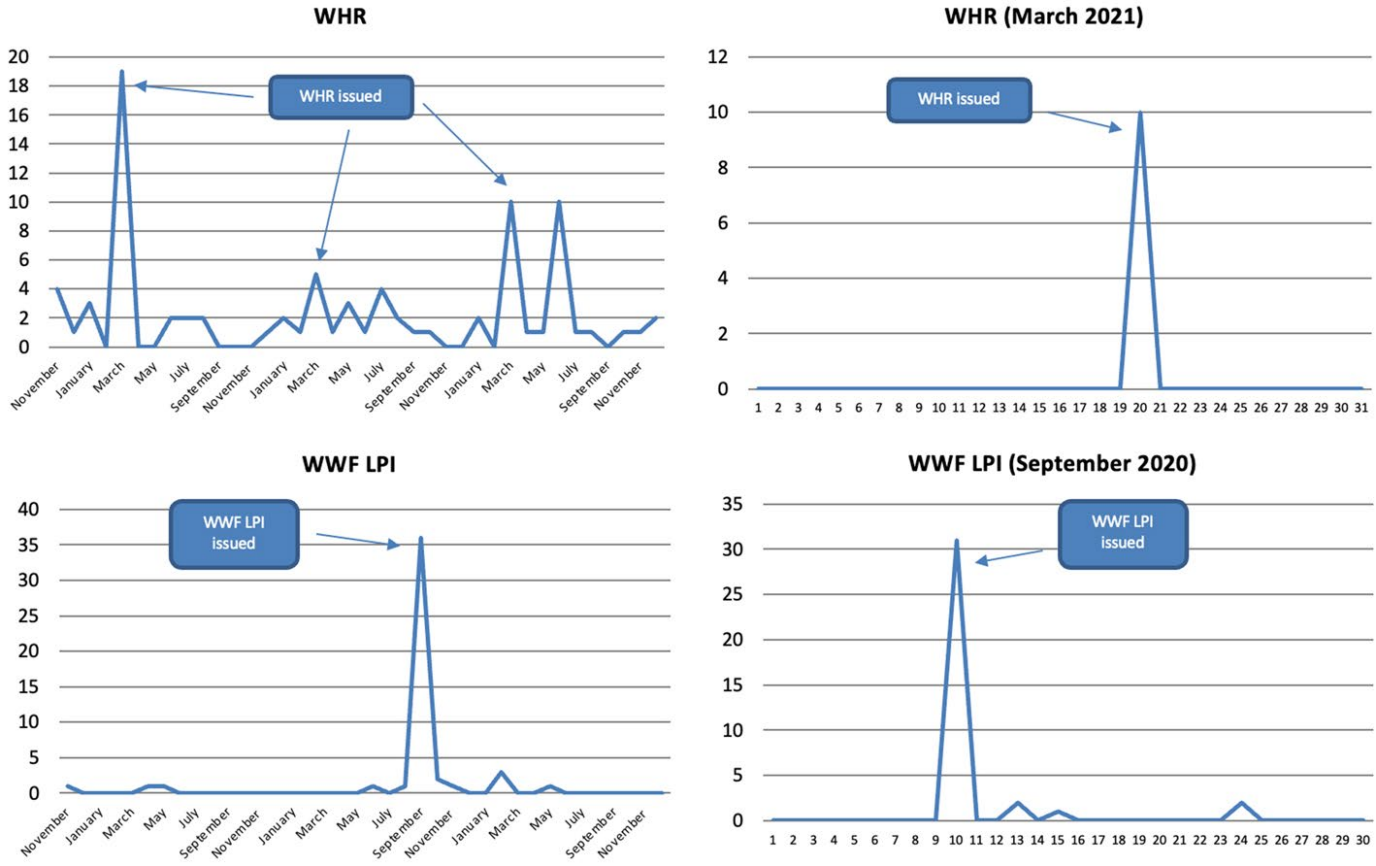
Number of references to selected well-being metrics on 32 Scottish and UK-wide radio stations and 13 Scottish and UK-wide TV channels by month (November 2018-December 2021) and by day (selected years and months), duplicates excluded. Source: TVEyes (author’s own elaboration)

The majority of mentions (54%) were made in just four months: November 2018, January and March 2019, and September 2020. These correspond to the months in which the 2018 GGCI, the 2019 Bank of Scotland QoLS, the 2019 WHR and the 2020 WWF LPI were issued, respectively. In fact, well-being metrics were mentioned again mostly, if not even only, in the months in which they got released. This trend can be seen clearly if we break down data by month for the top two metrics, which 47% of all mentions refer to (Fig. 8). All the peaks were reached when the metrics in question were issued (the only exception is the WHR, for which a peak can also be seen in June 2021). In fact, most mentions were once again made on the day in which these metrics were published, with some occasional coverage in the following days, after which they disappeared from the media landscape.

**Fig. 9:**
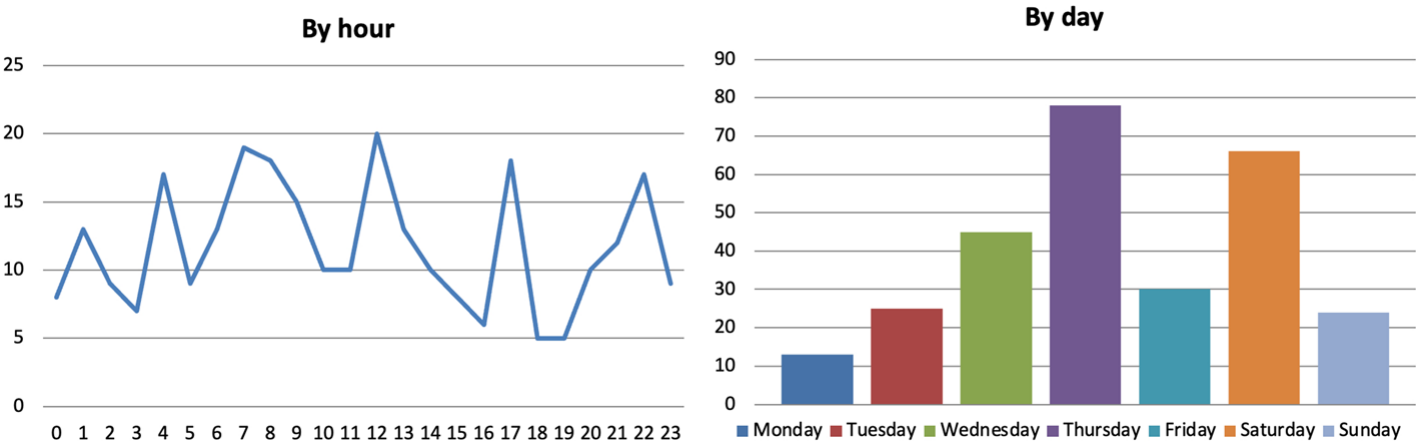
Number of references to 23 well-being metrics on 32 Scottish and UK-wide radio stations and 13 Scottish and UK-wide TV channels by hour and by day, November 2018-December 2021, duplicates excluded. Source: TVEyes (author’s own elaboration)

As for the time of the day in which mentions were made, there were five major peaks at 4 and 7 A.M. and at 12, 5 and 10 P.M. (Fig. 9). Only 15% of references were made during prime time (7–11 P.M.) as opposed to e.g. the early morning hours of 6–10 A.M. (23%). More mentions were made even between 4 and 8 A.M. than during prime time, although this is likely due to the re-airing of previous content. This shows the difficulty for well-being metrics to make it into the main evening schedules and suggests that well-being is a theme that is neither perceived to be worth reporting to a larger audience nor one that is raised frequently by policymakers participating in talk shows aired when most people are watching or listening. Data broken down by day also show that one third of mentions were made between Saturday and Sunday, although Thursday seems propitious, too.

**Fig. 10:**
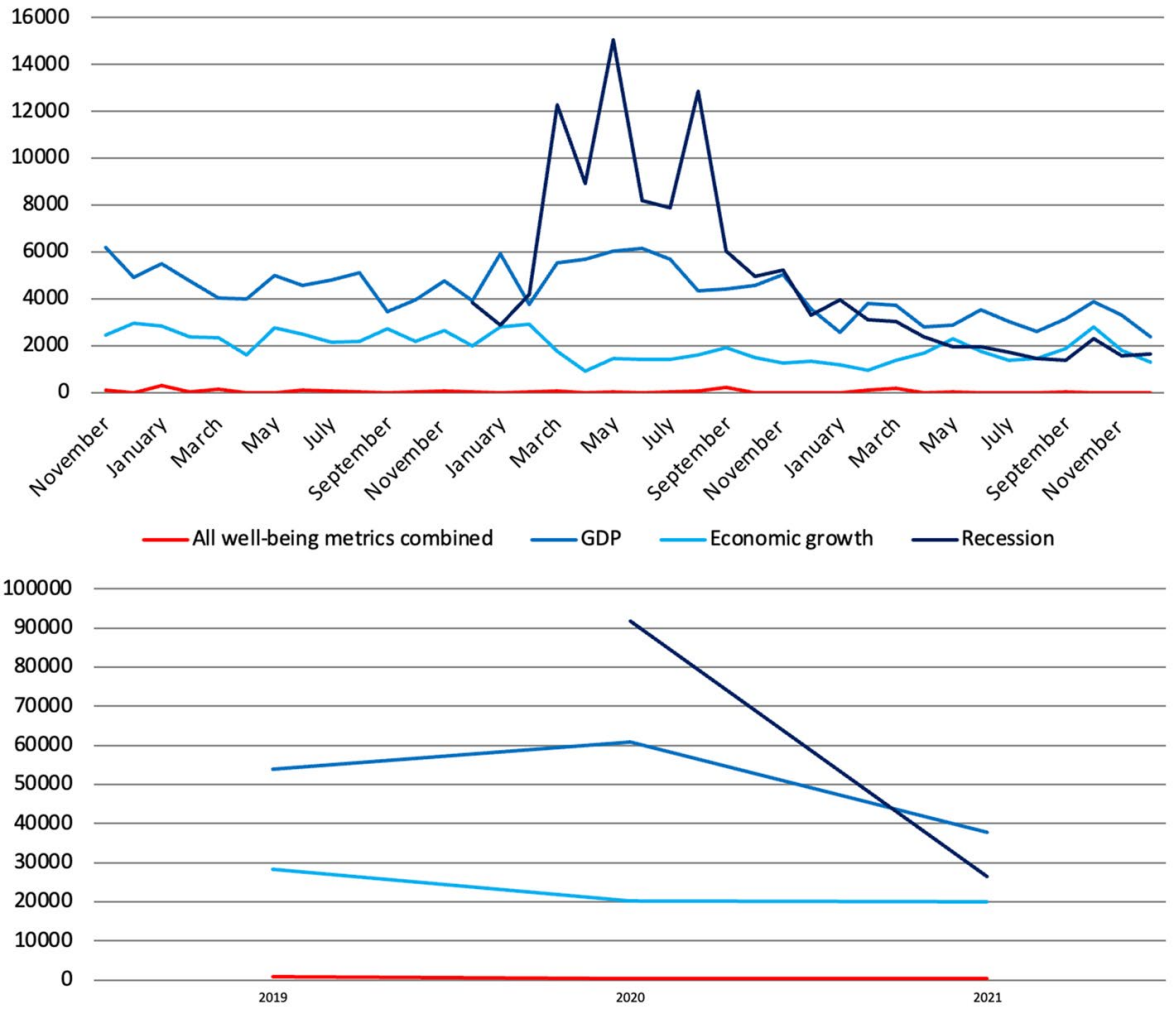
Number of references to 23 well-being metrics combined compared to GDP, economic growth and recession on 139 UK-wide and regional radio stations and 56 UK-wide and regional TV channels by month (November 2018-December 2021) and by year (2019–2021), duplicates included. Source: TVEyes (author’s own elaboration)

Finally, references to all well-being metrics combined are significantly lower than mentions of GDP, economic growth and recession (Fig. 10).^6^ What we also see clearly is the sharp increase in references to recession starting from March 2020 when the COVID-19 pandemic hit Europe and as the UK entered is first lockdown. While the reporting of well-being metrics was thus affected negatively, that of GDP and recession was instead affected positively. In 2021, references to all well-being metrics combined kept decreasing (halving compared to 2019), and so did mentions of GDP and recession which remained however 83 and 59 times as high, respectively, while references to economic growth stabilised. This suggests again that of particular concern especially in the first year of the pandemic was the impact this was going to have primarily in terms of and on GDP than in terms of and on well-being, concern that partially deflated in 2021, remaining however incomparably strong.

### Newspaper Coverage – Italy

**Fig. 11:**
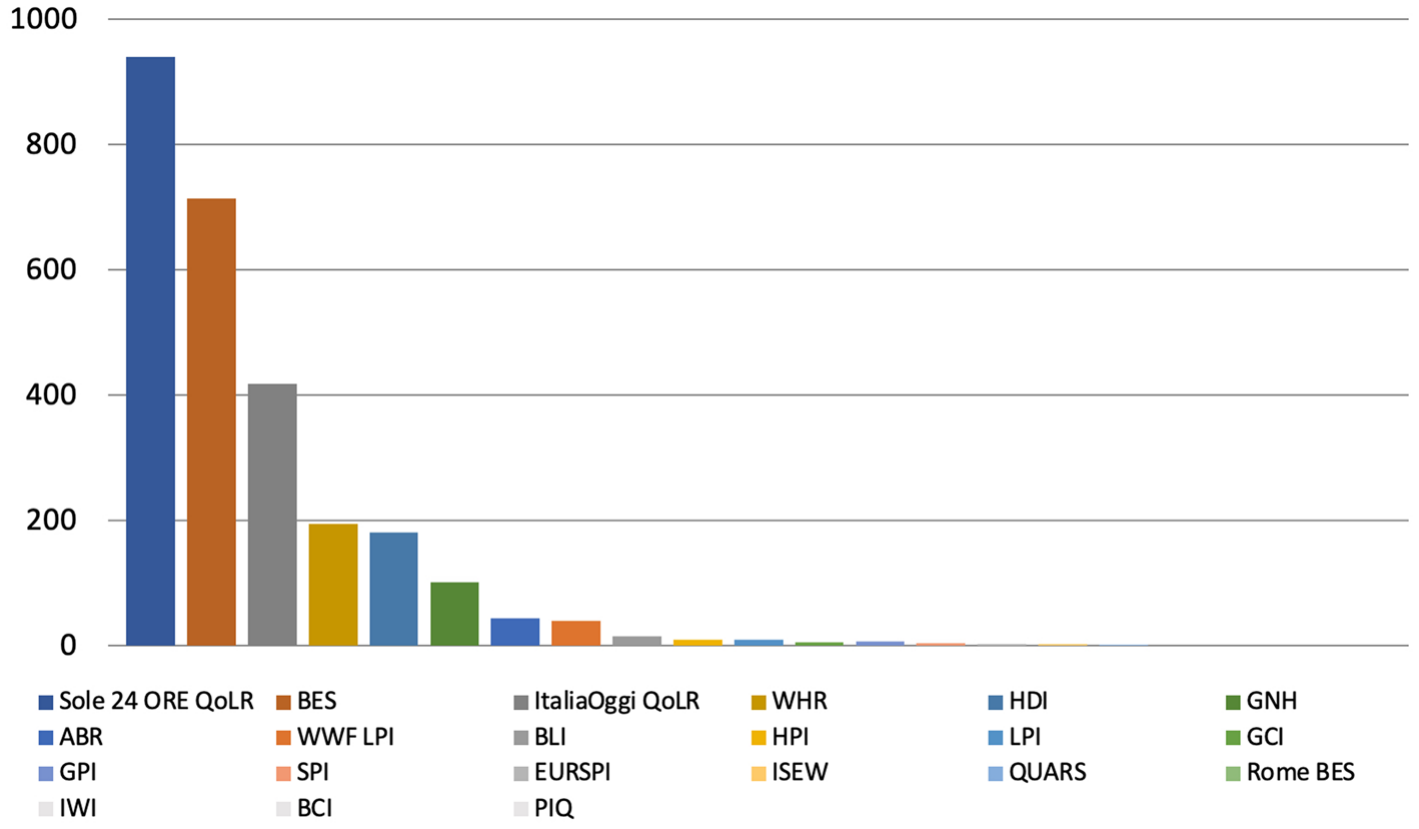
Number of articles published on 33 Italian newspapers mentioning at least once 21 well-being metrics by metric, 2017-2021, duplicates excluded. Source: Factiva (author’s own elaboration)

The metric that was found in the highest number of articles was the Sole 24 ORE QoLR (940), followed by the BES (713) and the ItaliaOggi QoLR (418), with the BCI, the IWI and the PIQ at the end of the scale (all of which returned 0 articles each), for a total of 2,685 articles (Fig. 11).

**Fig. 12:**
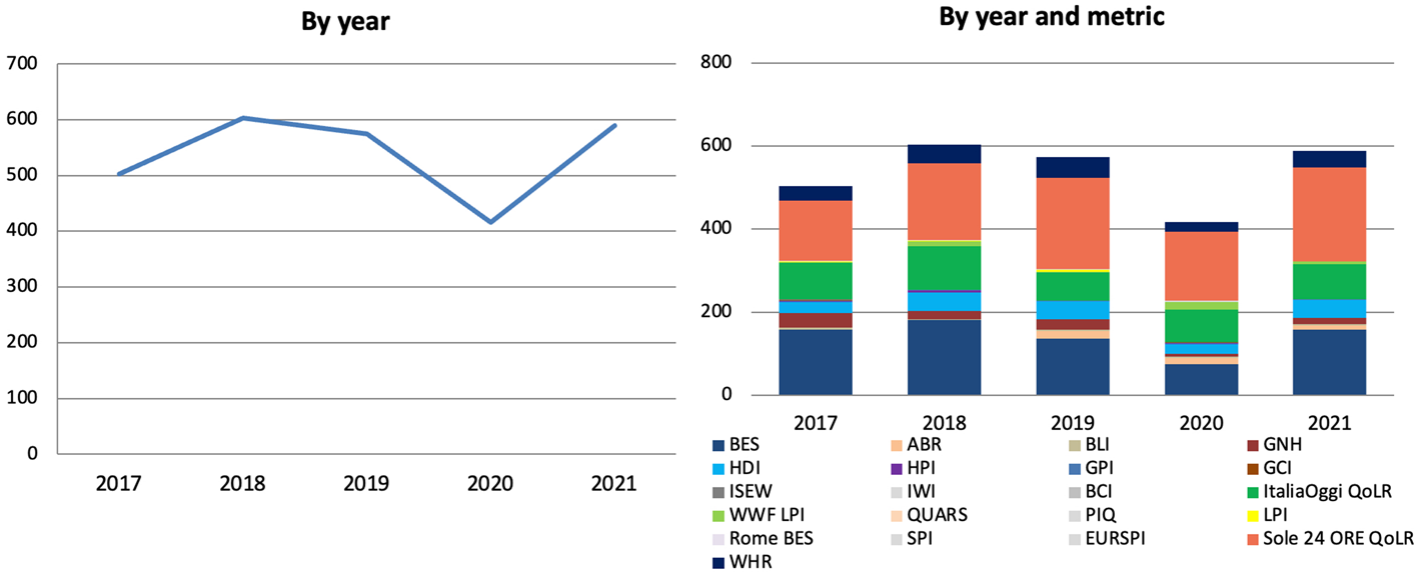
Number of articles published on 33 Italian newspapers mentioning at least once 21 well-being metrics by year and by year and metric, 2017–2021, duplicates excluded. Source: Factiva (author’s own elaboration)

The overall number of articles increased in 2018, decreased slightly the year after and more significantly by 28% in 2020 (Fig. 12). Since one metric was launched in 2018, that year’s increase may be due to that. However, the metric in question was the Rome BES, which was not found in any article, meaning the 2018 increase was unaffected by its launch. Such increase may have still been driven by the release of the WWF LPI (not iterated in 2017) but this was mentioned in only 12 articles, whereas the overall gap between 2017 and 2018 articles is much larger (100). As for the 2019 decrease, this was only limitedly due to the non-iteration of the WWF LPI (since this was again mentioned only in a handful of articles in 2018 upon its release) and occurred despite the launch of the ABR. While the 2018 increase cannot therefore be attributed to the release of new metrics or to the iteration of biannual ones but to a genuine increase in interest or perhaps to greater dissemination efforts from their promoters’ side, the 2019 decrease was conversely due to a decrease in the latter. As for the 2020 decrease, this may be due to the release of the 2020 BES being postponed to 2021. However, the number of articles that was published about the BES in previous years upon its release (22 on average) is much smaller than the number of articles missing (158). Moreover, such decrease occurred despite the release of the WWF LPI. Like in Scotland, the reduction in coverage in 2020 is therefore most likely attributable to the COVID-19 pandemic and the economic crisis that followed.^7^ In 2021, the total number of articles went back to its 2018 level. This, however, was mainly due to an increase in coverage of the BES (due to the above) and of the Sole 24 ORE QoLR (due mainly to the release of a sub-index in June, see below).

**Fig. 13:**
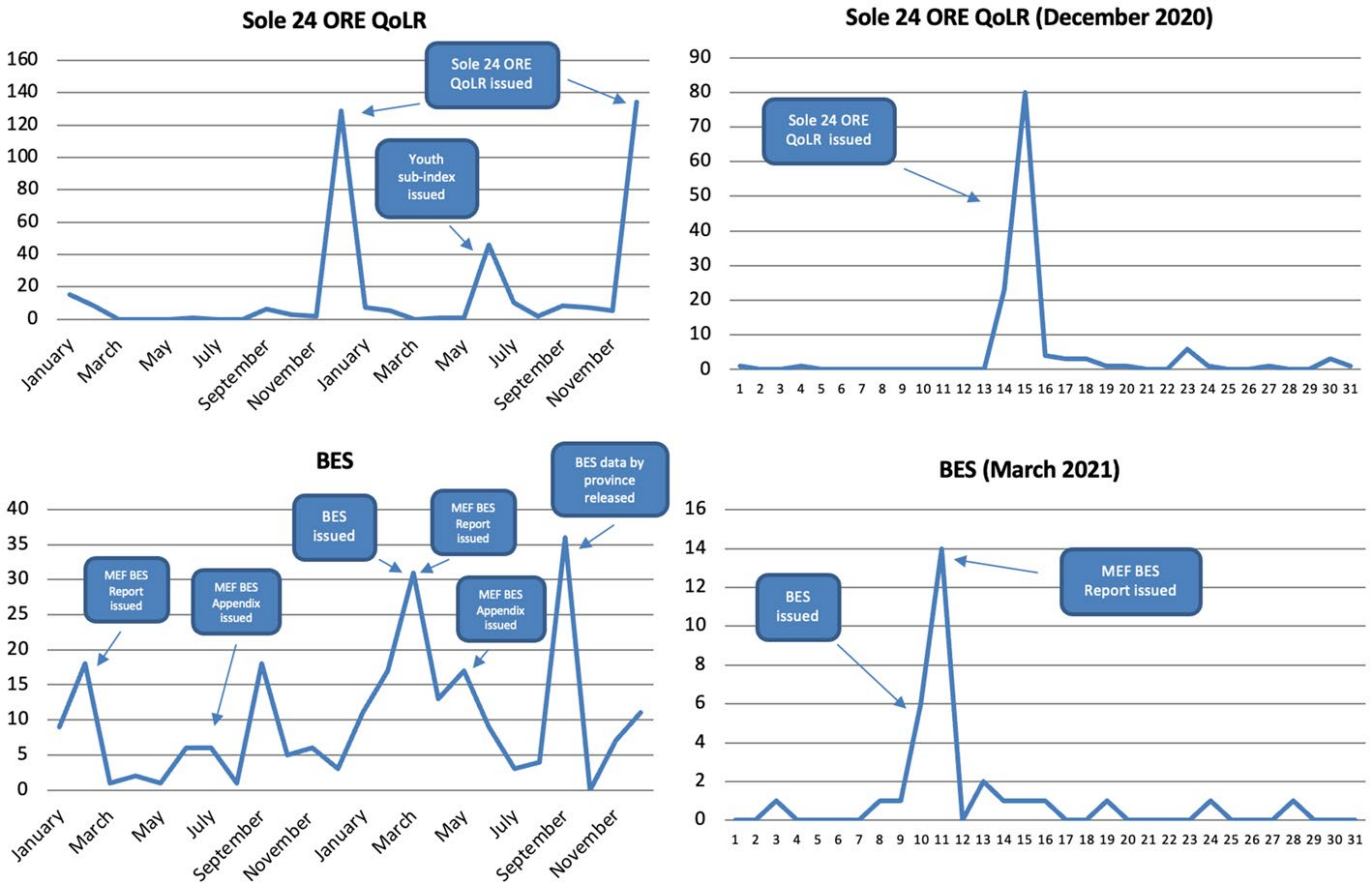
Number of articles published on 33 Italian newspapers mentioning at least once selected well-being metrics by month (2020–2021) and by day (selected years and months), duplicates excluded. Source: Factiva (author’s own elaboration)

Like in Scotland, the majority of articles were published in the months in which metrics were released and, within those months, almost exclusively on the day of their release. For instance, below is the number of articles sorted by month for the top two metrics, which 62% of all articles refer to, for the years of 2020 and 2021 (Fig. 13). The major peaks were reached in the months in which the metrics in question were issued. Most of the remaining peaks were made when related publications were released, as in June 2021 when *Sole 24 ORE* issued a Youth Quality of Life sub-index. The issuing of metrics and their reporting is so interlinked that in December 2020, when the 2020 BES was supposed to come out before being postponed to 2021, only a handful of articles mentioned it. In other words, not only did the BES get reported upon its release, but it also did *not* when *no* BES Report was issued. Note how the institutionalisation of the BES, and the consequent publication of yearly MEF BES Reports and Appendices, has granted it frequent coverage throughout the year compared to other metrics. In fact, of all metrics studied both in Italy and Scotland, the BES is the only one that was reported on a regular basis, so much so that between 2017 and 2021 it was mentioned in at least one article in every but one month, not to mention that in 2021 the MEF BES Report received greater coverage than ISTAT’s.

**Fig. 14:**
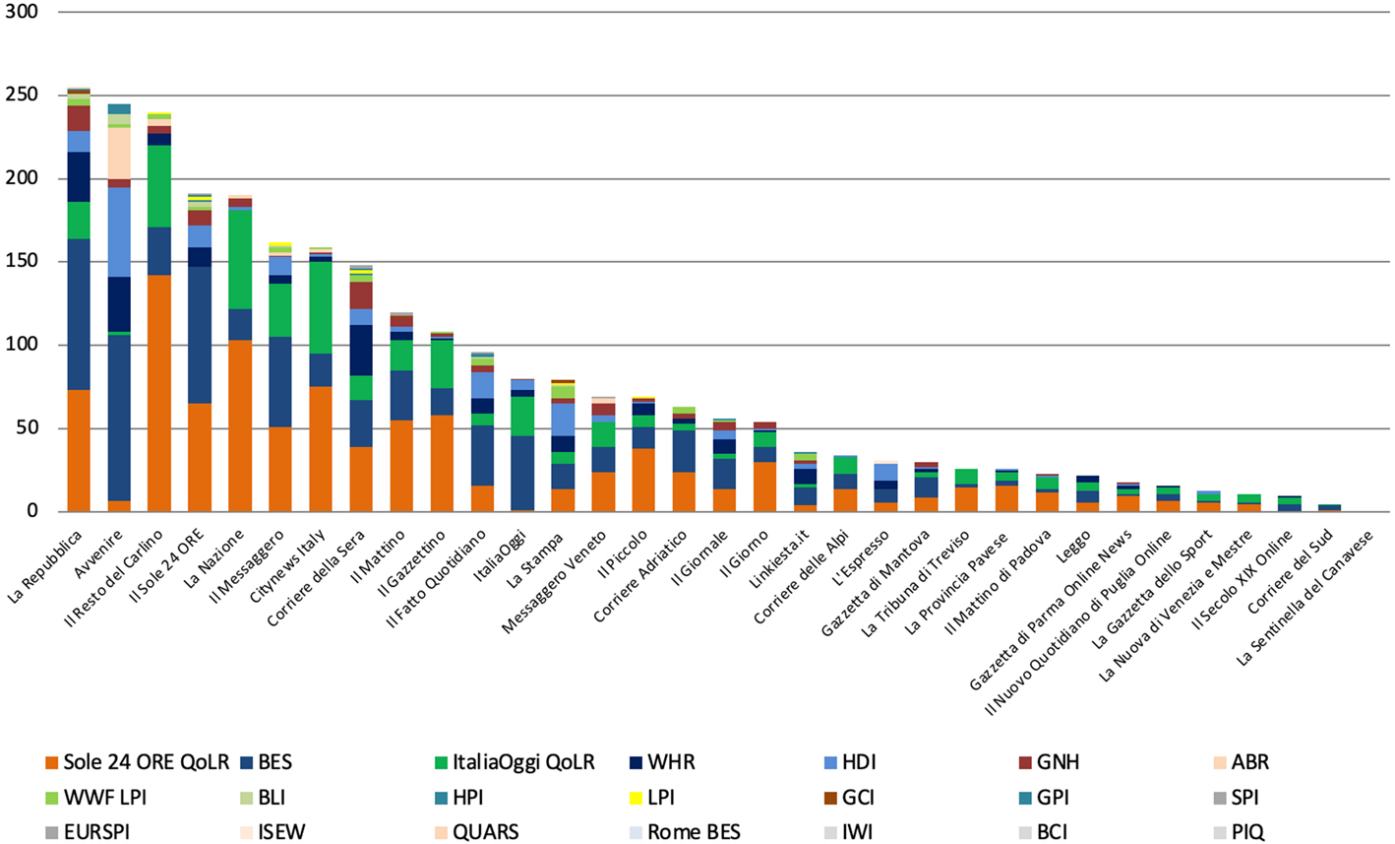
Number of articles published on 33 Italian newspapers mentioning at least once 21 well-being metrics by source and metric, 2017–2021, duplicates excluded. Source: Factiva (author’s own elaboration)

Half of all articles appeared in seven newspapers (Fig. 14). Unlike in Scotland where the sample of metrics was even larger, all sources but one published at least one article each and, among these, there was only one that published less than 10. However, metrics were reported differently between newspapers. For instance, *Sole 24 ORE*, *ItaliaOggi* and *Avvenire* (responsible for their homonymous rankings) almost never talked about their competitors’ metrics. Yet, they are among the top five newspapers in terms of references to the BES, despite this being a competitor. Interestingly, of all articles published by the first seven newspapers (in which again half of all articles appeared, and which are also among the most circulated in the country), only 6% are about the WHR and 3% about the GNH. The most read newspapers in the country thus did not seem interested in metrics that explicitly emphasise subjective indicators.

**Fig. 15:**
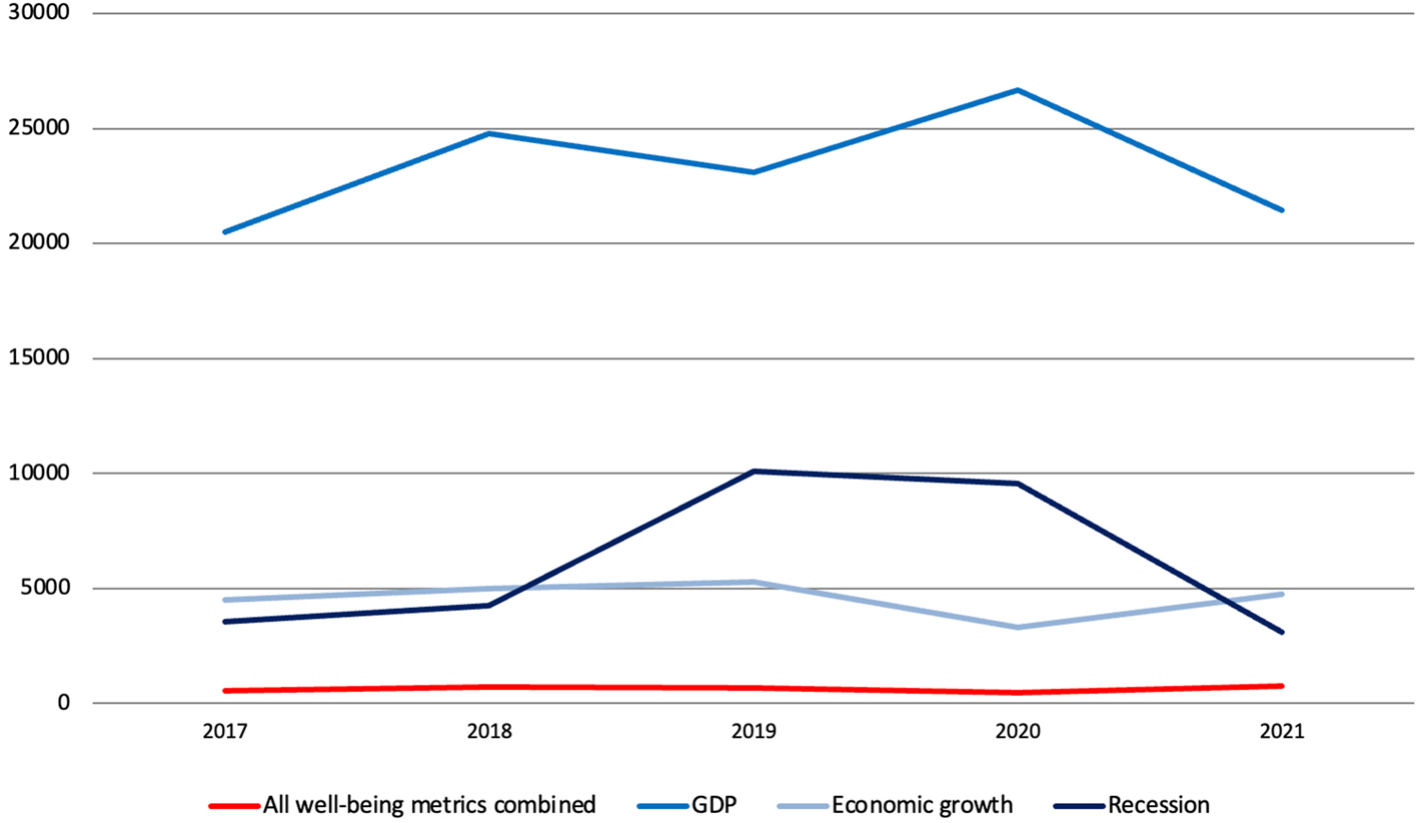
Number of articles published on 33 Italian newspapers mentioning at least once 21 well-being metrics combined compared to GDP, economic growth and recession by year, 2017–2021, duplicates included. Source: Factiva (author’s own elaboration)

The number of articles mentioning all 21 well-being metrics combined is 2.8% compared to the number of articles that mentioned at least once GDP over the same period, 14% compared to economic growth and 11% to recession (Fig. 15). Compared to 2019, the number of articles mentioning at least once GDP increased in 2020 by 16%, reaching the highest point in the period analysed. To this corresponded a 37% decrease in the number of articles covering economic growth and a slight decrease ( − 5%) in those mentioning recession, which however more than doubled compared to 2018 and almost tripled compared to 2017 (there was no big change compared to 2019 because in 2019 Italy had already entered a technical recession, hence the increase from 2018 to 2019). Once again, there is reason to believe that such changes are all connected to the COVID-19 pandemic and the economic crisis that followed. In 2021, articles concerning GDP and recession went back to their 2017 levels, and so did articles about economic growth which grew by 43%.

### Radio and TV Coverage – Italy

**Fig. 16:**
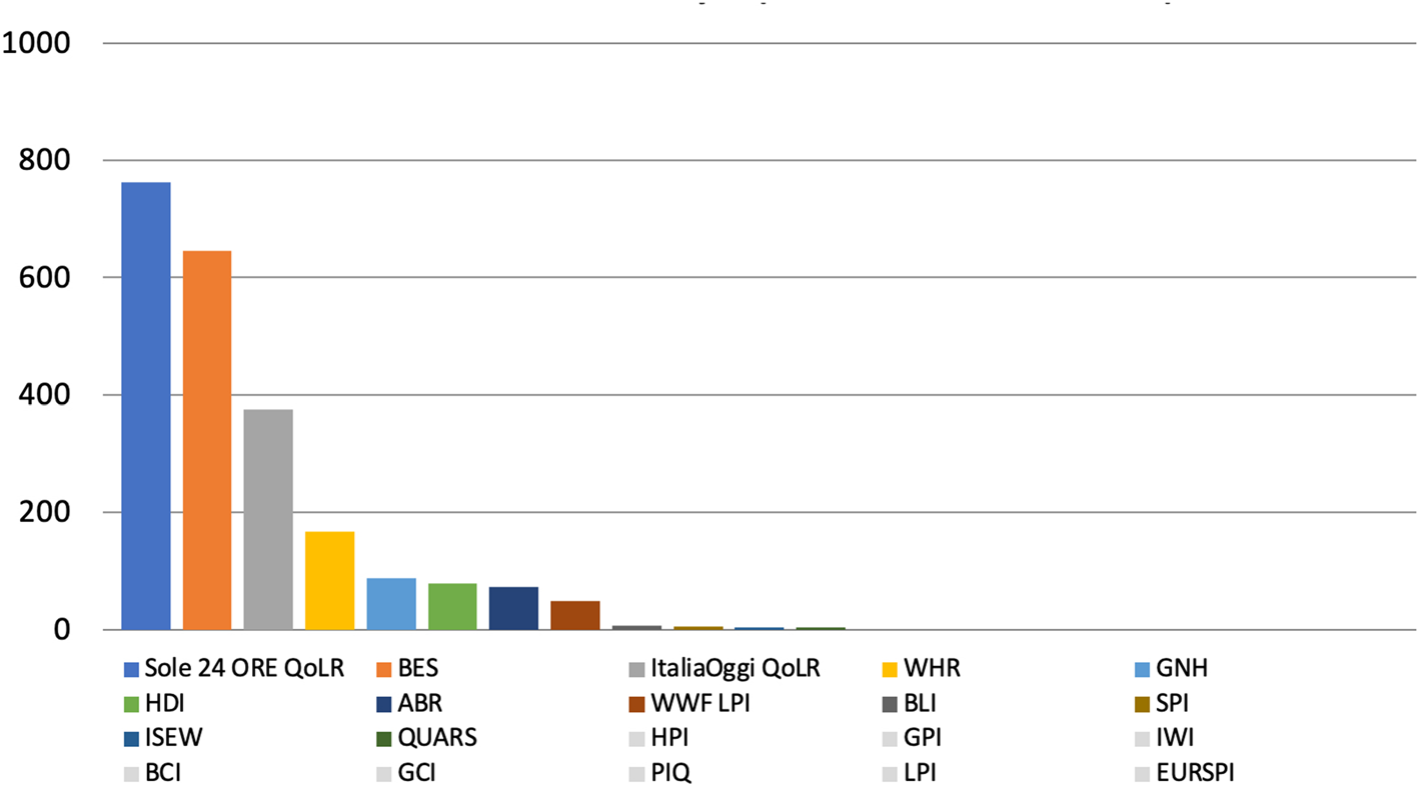
Number of references to 21 well-being metrics on 16 Italian radio and 21 Italian TV stations by metric, November 2018-December 2021, duplicates excluded. Source: TVEyes (author’s own elaboration)

The most mentioned metric over the 38-month period analysed was the Sole 24 ORE QoLR (with 762 references), followed by the BES (645) and the ItaliaOggi QoLR (375), with 9 metrics such LPI and the PIQ at the end of the scale (all of which returned 0 results each), for a total of 2,256 references (Fig. 16).

**Fig. 17:**
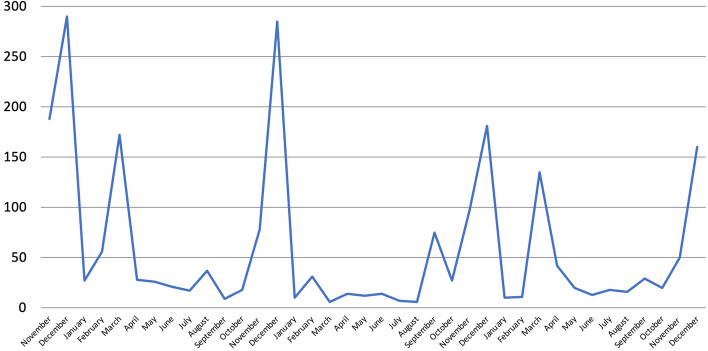
Number of references to 21 well-being metrics on 16 Italian radio stations and 21 Italian TV channels by month, November 2018-December 2021, duplicates excluded. Source: TVEyes (author’s own elaboration)

Overall references increased particularly in November and December 2018, March and December 2019, December 2020, and March and December 2021 (Fig. 17). Such peaks are all linked to the releases of three metrics: the BES, the ItaliaOggi QoLR and the Sole 24 ORE QoLR. Compared to 2019, overall mentions decreased by 38% in 2020. Metrics such as the BES ( − 69%), the Sole 24 ORE QoLR ( − 32%), the WHR ( − 60%), the GNH ( − 45%) and the HDI ( − 54%) all received less coverage in 2020 compared to 2019. As regards the BES, part of such decrease is due to the release of the 2020 BES being delayed to 2021. However, even if we add references to the BES made in March 2021 (when this was published) to 2020, these would still be 41% lower. Such large decrease in total coverage occurred despite the release of the WWF LPI, whose number of mentions increased instead significantly (+ 1,300%) due however to this not being issued in 2019. As argued previously, there is reason to believe that such overall contraction, which is not therefore attributable to changes in the release of metrics and in fact occurred despite the issuing of biannual metrics, was due to the COVID-19 pandemic and the economic crisis that followed, whose impact could also be seen in 2021. In fact, while overall references increased by 10% in 2021, compared to 2019 these were still 32% lower. Furthermore, such increase is mainly due to the BES receiving greater coverage due to its release being postponed to 2021. If references to the BES made in March 2021 are thus added to 2020, and if the highest number of mentions this received upon its publication before the pandemic is added to 2021, overall references would still decrease by 27% in 2020 compared to 2019 and by 11% in 2021 compared to 2020. In other words, had BES Reports been published in both 2020 and 2021 as in previous years, the number of overall references would have most likely decreased in 2021, too. Lastly, it is worth mentioning that no single metric was reported at least once every single month. However, the BES was again very close, with mentions found in all but three. Interestingly, the 2019 HDI got almost no coverage, whereas the 2020 one (released in December 2020 for its 30th anniversary) did not get any at all, exactly like in Scotland.

**Fig. 18:**
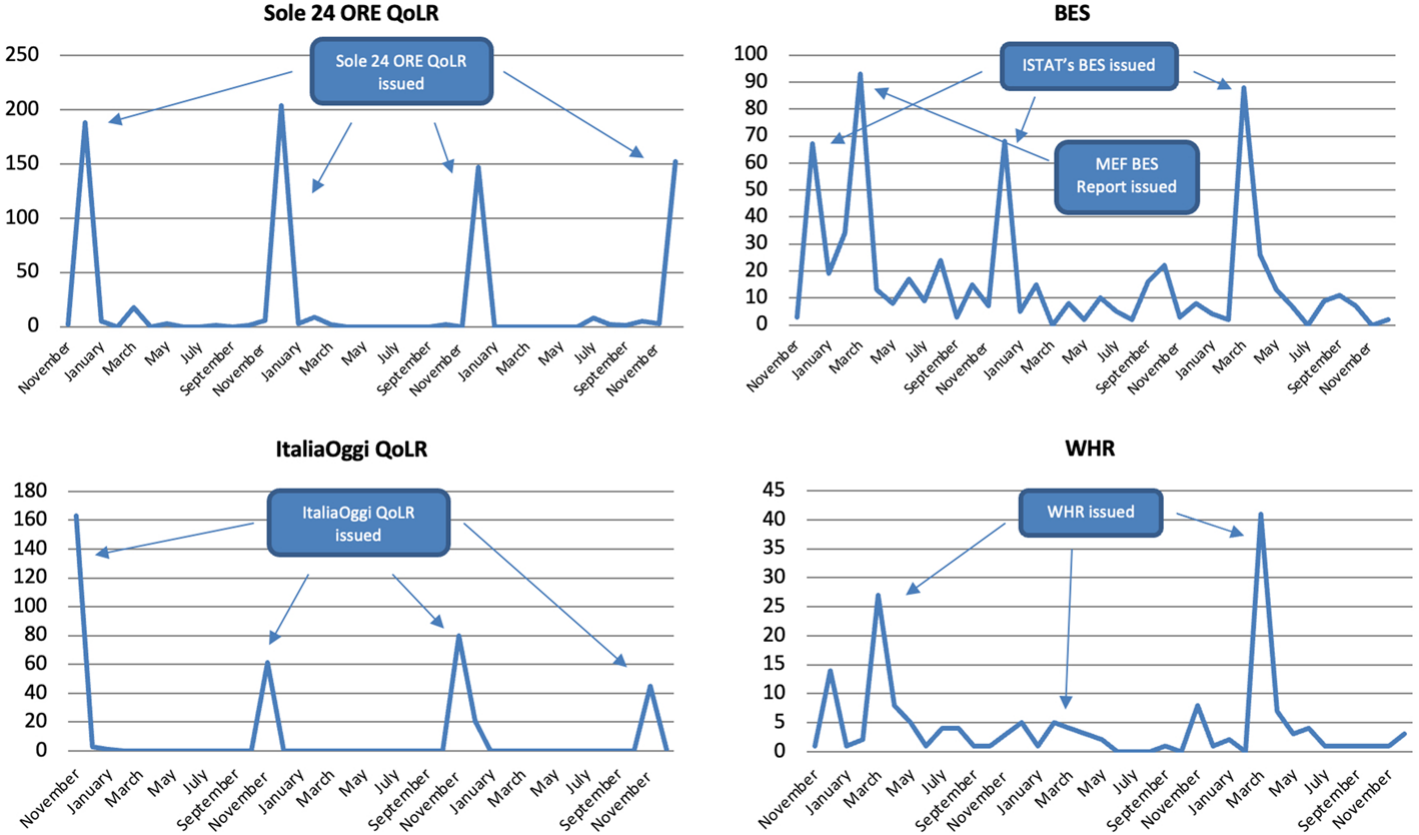
Number of references to selected well-being metrics on 16 Italian radio stations and 21 Italian TV channels by month, November 2018-December 2021, duplicates excluded. Source: TVEyes (author’s own elaboration)

The majority of references (68%) were made in the months in which metrics were issued (except for those that are not updated regularly). Above, for instance, are references to the top four metrics sorted by month (Fig. 18). All the highest peaks were reached when the metrics in question were released. The BES, for instance, received greater coverage when the 2018, 2019 and 2020 ISTAT BES Reports and the 2019 MEF BES Report were issued,^8^ with half of all mentions made in just these months. In the case of the ItaliaOggi and Sole 24 ORE QoLRs this trend was even more evident, with both getting covered almost exclusively in the months of their releases.

**Fig. 19:**
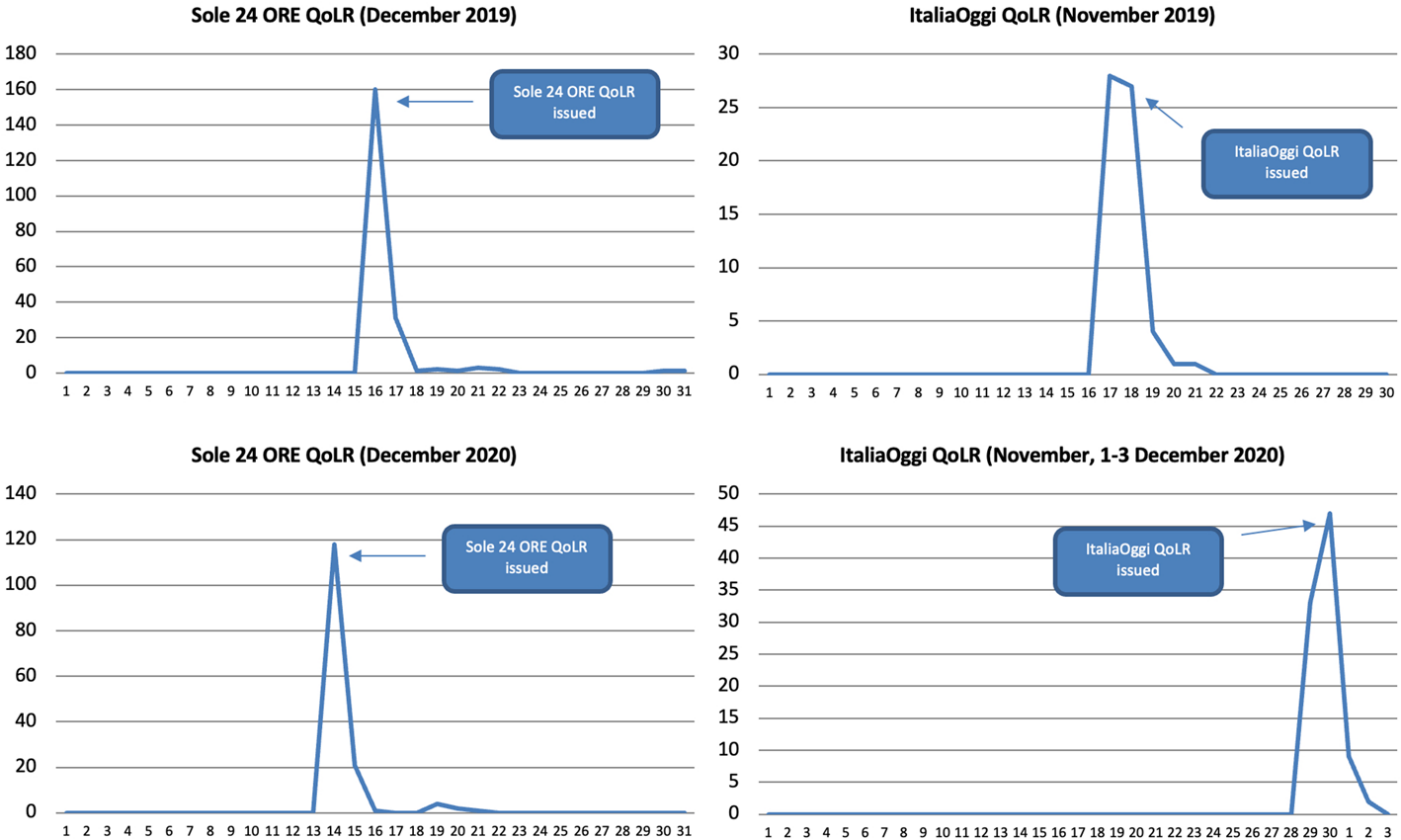
Number of references to selected well-being metrics on 16 Italian radio stations and 21 Italian TV channels by day, selected years and months, duplicates excluded. Source: TVEyes (author’s own elaboration)

The majority of references within the above months were made on the days these metrics were issued (Fig. 19). Above, for instance, are 2019 and 2020 references to the Sole 24 ORE and ItaliaOggi QoLRs sorted by day for their release month. None of them was reported in the weeks preceding their release; their coverage grew sharply on the day of their issuing (in all four cases a Monday); and their coverage plummeted drastically after one or two days, after which it flattened out. The ItaliaOggi QoLR actually started to be covered on Sunday already (the same happened in 2018 and 2021) but this may simply be due to its editors sending press releases in advance that are not embargoed as a way to increase interest in the Monday release.

**Fig. 20:**
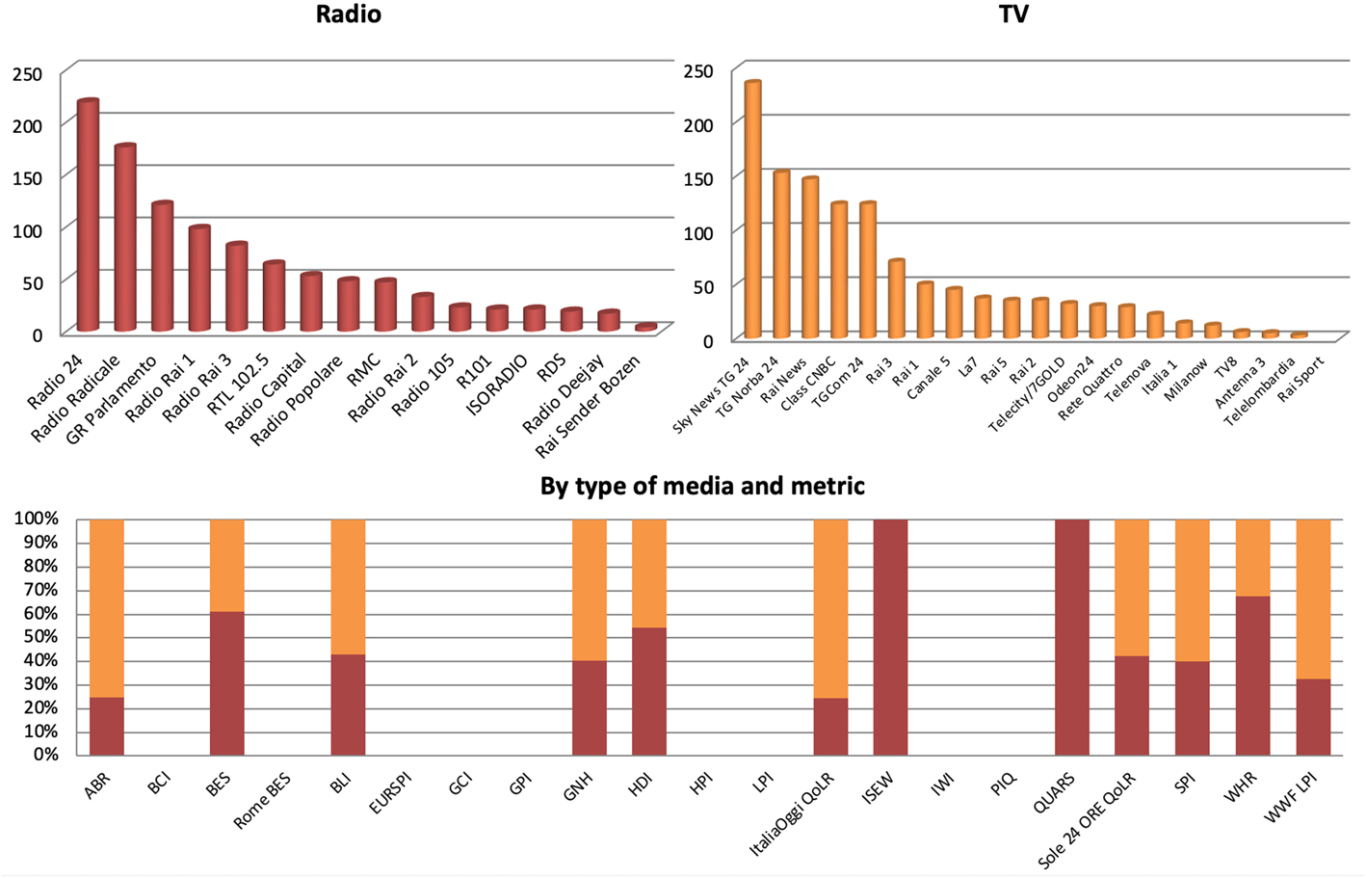
Number of references to 21 well-being metrics on 16 Italian radio stations and 21 Italian TV channels by type of media and by type of media and metric, November 2018-December 2021, duplicates excluded. Source: TVEyes (author’s own elaboration)

Slightly more than half of all references (54%) were made on TV (Fig. 20). As for radio, half of all references were made on just three stations, all of them sources that specialise in politics and current affairs, whereas only 23% were made on the more “commercial” stations included in the sample (*RTL 102.5*, *Radio Capital*, *RMC*, *Radio 105*, *R101*, *RDS* and *Radio Deejay*). As for TV, the trend is similar, in the sense that the top five sources, which account for 65% of all TV mentions, are all-news channels. Breaking down data further by type of outlet, metric and source (not shown here) reveals again an interesting competition between *Class CNBC* (owned by Class Editori, the same company that owns *ItaliaOggi*) and *Radio 24* (owned by Confindustria, which also owns *Sole 24 ORE*), with both almost completely ignoring each other’s ranking. This shows the difference between commercial, newspaper rankings and official, institutionalised metrics such as the BES. In fact, despite the above and the BES being again a competitor, both were among the sources on which the BES was mentioned the most. Another finding worth noting is that metrics that more explicitly emphasise subjective indicators such as the GNH and the WHR were not mentioned frequently on “news and talk” or less commercial sources. Indeed, of all mentions made on the first five radio stations and TV channels (which account for 67% of all mentions in the sample and are again mainly specialised sources or all-news channels), less than 5% were about the WHR and only 1% about the GNH. This suggests that these metrics are not being taken seriously by the journalists working for these sources or by the policymakers interviewed by them.

**Fig. 21:**
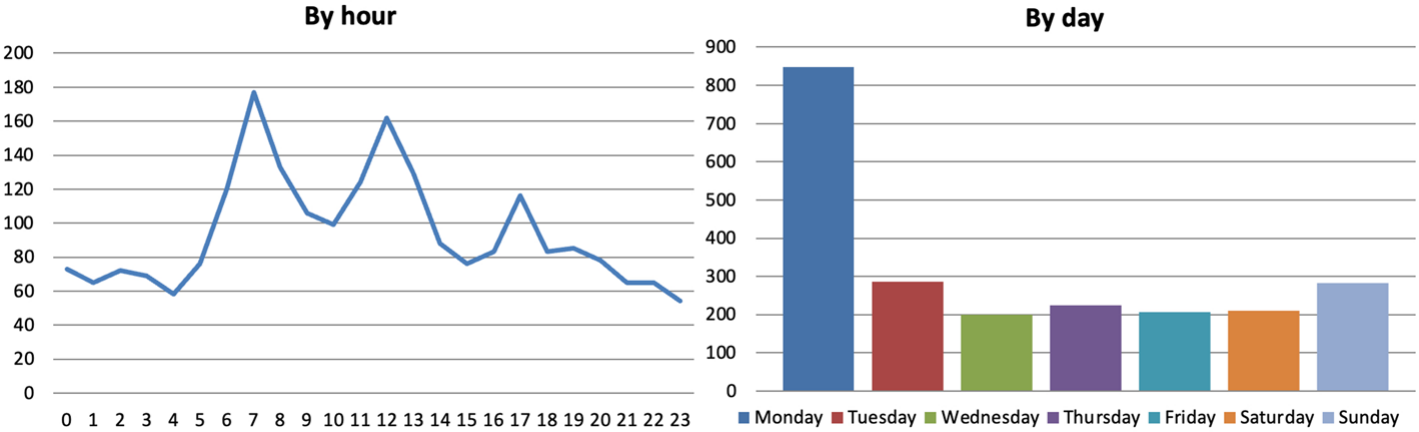
Number of references to 21 well-being metrics on 16 Italian radio stations and 21 Italian TV channels by hour and by day, November 2018-December 2021, duplicates excluded. Source: TVEyes (author’s own elaboration)

As for the time of the day in which mentions were made, similarly to Scotland there were three peaks at 7 A.M., 12 P.M. and 5 P.M. (Fig. 21). Also similarly to Scotland, only 9% of references were made during prime time (8–11 P.M.) as opposed to e.g. the early morning hours of 6–9 A.M. (19%) or between 10 A.M. and 1 P.M. (17%). This suggests that well-being is a theme that is neither perceived to be worth reporting to a larger audience nor one that is raised frequently by policymakers while participating in talk shows aired during prime time. Data broken down by day also show that about 40% of mentions were made on Mondays. This is due to Mondays being a day in which ‘there are no big news’ in which it is easier to talk about ‘low-salient issues’, a TV journalist explained to me (personal communication). Indeed, although it is true that the results above are due to Monday being the day the ItaliaOggi and the Sole 24 ORE QoLRs were released, the fact that these were and keep being released on Mondays is probably exactly for this reason.

**Fig. 22:**
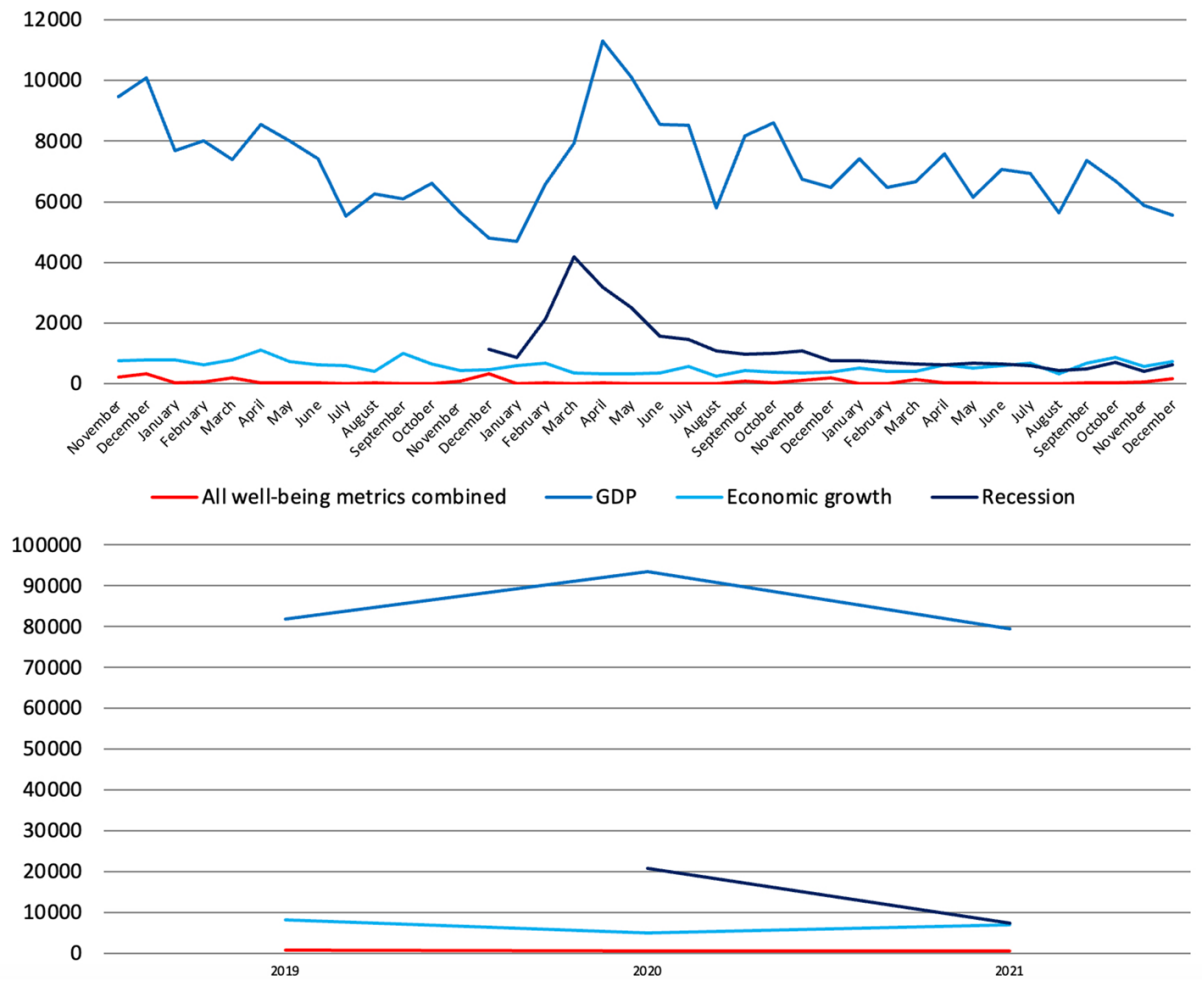
Number of references to 21 well-being metrics combined compared to GDP, economic growth and recession on 16 Italian radio stations and 21 Italian TV channels by month (November 2018-December 2021) and by year (2019–2021), duplicates included. Source: TVEyes (author’s own elaboration)

Finally, above is the number of references to all 21 metrics combined compared to that of GDP, economic growth and recession (Fig. 22). Data sorted by month show that there was a sharp increase in references to GDP and recession starting from February 2020 as soon as the COVID-19 pandemic hit the country, with the former increasing by 11,549 units (+14%) in 2020 compared to 2019, whereas references to all well-being metrics combined decreased once again by more than a third. While the reporting of well-being metrics was thus affected negatively, that of GDP and related queries such as recession was affected positively instead. In other words, this suggest again that of particular concern during the pandemic was the impact this was having and going to have primarily on GDP and in terms of output than in terms of and on well-being. In 2021, references to GDP returned to their pre-pandemic level, and so did those to economic growth (which increased by 39%) and recession, at least from what December 2019 data suggest. References to all well-being metrics combined increased slightly, due again however to changes in the year of release of the BES.

## Discussion

Findings revealed almost the exact same reporting patterns in both countries. Well-being metrics were mentioned occasionally, particularly during weekends in Scotland and on Mondays in Italy and mainly upon their publication in line with Morse (2011b, 2013). So much so that when this was postponed, their reporting would be postponed, too. On the one hand, this is not surprising. After all, journalists report news and, once the new results of a metric have been reported, these are not news anymore. On the other hand, GDP figures are not released daily yet it – and related concepts such as economic growth and recession – *do* get mentioned regularly and with a striking frequency.^9^ Well-being metrics are thus occasional news items that get covered mainly on a passive basis under the stimuli of their producers through the use of press releases and when there seems to be a news vacuum, whereas GDP is an ordinary component of both countries’ public debate that is constantly mentioned proactively.

In fact, what was similar in both countries was also the density and volume of coverage of all well-being metrics combined compared to that of GDP. In the case of newspapers, for instance, the former was 1.6% in Scotland and 2.8% in Italy. This is similar to what Morse (2013) found. And while his methodology was unrobust, findings do support his claim about that of well-being metrics being ‘hardly […] an extensive coverage’ (2013, p. 247), especially when we consider that we are comparing the density of reporting of more than twenty metrics *with that of just one*. This also confirms claims made in the literature about the overexposure of GDP (Fioramonti, 2013) and the lack of coverage of well-being metrics (European Commission, 2013).

Yet, journalists do not only re-post press releases or discuss what they are interested in but also constantly report or broadcast what policymakers say. So, if coverage of well-being metrics has been overall low and lower during prime time, that is also due to *policymakers* not referring to them when speaking to journalists, in public, or when larger audiences are tuned. Likewise, if coverage of GDP and economic growth is high that is due to *policymakers* referring to them constantly. And, since what policymakers talk or do not talk about in public reflects inevitably their priorities and what is or what is not on the political agenda, this suggests that well-being is still not a priority contrarily instead to the pursuit of economic growth. This is in line with previous studies that investigated the factors inhibiting the use and impact of well-being metrics which showed a prevailing view of economic growth as a prerequisite for well-being and therefore widespread support for it (Hayden & Wilson, 2016, 2017, 2018a, b; Seaford, 2013; Thiry et al., 2013).

Indeed, the economic crisis that followed the COVID-19 pandemic did not generate any increase in the media reporting of well-being metrics which decreased in both countries, with coverage of GDP and related queries increasing instead.^10^ Economic crises therefore favour the status quo, depressing rather than fostering the use and impact of well-being metrics. Had the crisis been an occasion to ‘build back better’ (OECD, 2020, p. 2) and provided ‘a time of awakening’ (Gills, 2020, p. 578), the use of and interest in well-being metrics would have not decreased – *it would have increased and been strengthened.* After all, *if not now, then when?* Yet, the opposite happened. Between the 1970s and 1980s, interest in social indicators was negatively affected by a then precarious economic situation which ‘restored prominence […] to GDP as the main monitoring tool’ (Fleurbaey & Blanchet, 2013, p. 5). Most recently, Giovannini (2020, n/a) claimed that ‘before the 2008–09 crisis, at the OECD we tried to persuade lots of governments to adopt a “beyond GDP” thinking […]. The crisis killed that discussion’. As they say, history repeats itself.

What differed instead between the two countries is the classification of well-being metrics in terms of their levels of coverage (due to differences in the metrics included in each country’s sample and to the fact that, in Italy, the developers of some metrics also owned some of the outlets searched), the periods in which these got covered (due to the above but also to the different political and news cycles of the countries in question), but above all their overall level of coverage. In the case of newspapers, 1,015 articles were published on Scottish and UK-wide sources (almost exclusively on the latter) whereas 2,685 in Italy (the Italian sample included two more sources, but that does not justify such a large gap, not to mention that the actual number of newspapers searched was lower and that the sample of metrics for Scotland was larger). In the case of radio and TV, 281 mentions were made on Scottish and UK-wide sources, whereas 2,256 in Italy, despite the Italian sample including eight fewer sources. Italy’s much higher coverage, combined with the higher number of newspapers that showed interest in well-being metrics and Italy’s longer and more established history of well-being measurement show, from a comparative perspective, that Italy is more advanced than Scotland as far as the promotion of the well-being agenda is concerned. Conversely, Scotland’s coverage levels show that Heins and Pautz (2021, p. 100) were right in claiming that a ‘surprising dearth of media interest characterises the Scottish wellbeing debate’.

Surprisingly, most composite indices (such as the HPI, the SPI, the IWI and the HKI, to name but a few) were almost if not even fully ignored by journalists, whereas those metrics that lack an overall composite index such as the BES were among the ones that were picked up the most. It is often believed (see Wallace & Schmuecker, 2012) that composite indices will help obtain greater media coverage and achieve in turn greater policy change. However, findings show that the way a metric is presented is much less important, if at all, than its officiality and the authority of its developer. This explains how in less than a decade the BES managed to become the second most mentioned metric in Italy (just below the Sole 24 ORE QoLR, a 32-year-old publication, and above the ItaliaOggi QoLR, a 23-year-old one), the only metric to be mentioned heterogeneously throughout the year and to be so especially by *Sole 24 ORE* and *Radio 24*, and *ItaliaOggi* and *Class CNBC* despite these not talking about each other’s ranking. This seems to apply globally, too: it does not seem a coincidence that the WHR and the HDI, both United Nations outputs, have gained much more traction than the SPI, the HPI, the LPI and so on.

Nonetheless, being overseen by an independent body is just as crucial. This can be seen clearly in the different levels of media coverage received by the two countries’ official frameworks, with the BES, which returned 1,358 results between articles and mentions, ranking again as the second most referenced metric in Italy, and the NPF, which returned only 35, ranking as one of the least mentioned metrics in Scotland. The reason for such large gap lies in the different ownership of the two frameworks. Since the publication of the first BES Report in March 2013, there have been eight different governments in Italy supported by different majorities. Yet, this has not impacted on the promotion of the BES since this is independently overseen and promoted by ISTAT. In Scotland, there have been five governments since the launch of the NPF in November 2007, all led by the SNP (with the recent addiction of the Scottish Green Party). Yet despite the higher number of years and the lower number of governments, the continued lack of attention towards the promotion of the NPF from the SNP-led government, whom the framework effectively belongs to, has determined its continued absence from the media landscape.^11^

It may be argued that journalists and policymakers will have not referred to specific metrics but to individual indicators within them. This is certainly true. However, if, say, a journalist cited an indicator from the NPF without mentioning the latter, that would be impossible for anyone to find out. Moreover, and most importantly, if they only cited one or a handful then the framework *as a whole* will have been in a way useless, as almost all the indicators included in the NPF already existed before its creation and the point of having them together is *exactly* to highlight the interconnectedness of different policy areas. The Scottish First Minister herself noted that, pointing out that ‘the National Performance Framework is intended to be a cross-cutting framework and it is important not to see anything that we capture in isolation’ (Sturgeon, 2021, n/a).

As far as newspaper coverage is concerned, the number of overall mentions is missing; no sentiment analysis was conducted; and coverage figures of GDP and related terms include duplicates (this last point also applies to radio and TV coverage). Regarding the first issue, this cannot be solved unless significant funding is available to cover the large amount of time needed to go through each article. Regarding the second issue, if articles about GDP were largely critical then during the COVID-19 pandemic and the economic crisis that followed those mentioning well-being metrics should have increased and those mentioning GDP and related terms decreased, whereas the opposite happened. In a way, then, the COVID-19 pandemic *did* enable me to perform a sentiment analysis. Regarding the third issue, figures do include duplicates but exclude cases in which references were made using other expressions which are much more commonly used, so it is likely that on balance they will be fairly accurate, *if not even an underestimation*.

## Conclusions

This article provided an in-depth investigation of media coverage of well-being metrics in Scotland and Italy on which no data were previously available. In fact, to the best of my knowledge no such data have ever been made available for any other country despite the key role that the media are believed to play for the success of the well-being agenda. It is true that studies were conducted about newspaper coverage (Bassi, et al., 2011; Hák et al., 2012; Morse, 2011a, b; 2013; 2015; 2016; 2018), but the methodology employed was unrobust and findings unreliable. This study therefore substantiated for the first time claims made in the literature about the overexposure of GDP (Fioramonti, 2013) and the lack of coverage of well-being metrics (European Commission, 2013). Moreover, media reporting tools such as TVEyes have been rarely used in academia, so this study also contributed to exploring the use of new research tools. The breakout of the COVID-19 pandemic also enabled me to provide the first analysis of media coverage of well-being metrics before and during an economic crisis, which other studies will not be able to conduct, at least with reference to this crisis, given that 2020 and 2021 radio and TV transcripts have already been deleted from TVEyes.

What, then, could increase the media coverage of well-being metrics? First, more effort should be put into promoting the well-being agenda when the economy is expanding as opposed to periods in which growth is lacking. This is because in such cases the attention will predominantly be on how to put the economy back on a growth track because *that* is the problem. Second, expectations around composite indices should be reconsidered and more effort should be put into developing official, national and independent frameworks. Due indeed to their limited national relevance, global metrics generate mediocre to no coverage at all and in any case only upon their release, showing the ability of their promoters to craft good press releases rather than the capacity to spark a debate throughout the year which only the institutionalisation of an official, national, independent framework like the BES has shown to be capable of. Third, press releases should be sent considering the day and time journalists are more receptive and when the news cycle is more propitious. Data from both countries show that well-being metrics were mentioned particularly in the early morning, at lunchtime and in the afternoon, but not much during prime time. They also show that, as far as radio and TV are concerned, they get reported especially during weekends in Scotland and on Mondays in Italy, due to there being a sort of news vacuum in these days. Until well-being metrics gain currency and saliency, exploiting these days and times is going to be the most impactful strategy.

## Appendix 1 − Notes about the use of Factiva and TVEyes

### Factiva

Simple and intuitive at first glance, Factiva is much more complicated to use than one may think. There are indeed six issues one needs to address to avoid misinterpreting data:

1. The coverage start date of sources does not match their launch date but varies depending on when agreements between Factiva and publishers were signed. This means that, for any given query, the number of articles returned will always at some point increase not because of an increase in interest but because of an increase in the sources covered;
2. Conversely, a decrease in articles might reflect a discontinuation, i.e. be due to said agreements expiring;
3. Increases or decreases in articles may also be due to more sources starting/ceasing naturally to be published;
4. Some sources are covered fully, others partially and for others still only abstracts are available (see Dow Jones, 2012). This means that increases/decreases in articles might be due not to increases/decreases in interest but to some sources being fully/partially covered;
5. For many sources Factiva reports delivery problems, so decreases in articles might be due to suspended deliveries;
6. Finally, sometimes Factiva includes sources in the wrong category. For instance, among Italian newspapers it includes both *ForeignAffairs.co.nz* (whose country of origin is New Zealand) and *News Bites* (Australia), meaning that increases in articles may originate from irrelevant sources.

To solve these issues, I manually collected information about all Italian, Scottish and UK-wide^12^ sources (590 in total). I then used this list to exclude any sources which were not newspapers and to determine which ones to include in my sample and the start year of my searches (for a list see Appendix 2). As for Scotland, I included only sources with full coverage agreements that were not discontinued by the end of 2021. Since all metrics but one had been launched by 2017, I chose 2017 as the start year of my search and, therefore, December 31, 2016, as the latest date sources could have been added to the database for these to be considered. The final sample included 56 sources (27 Scottish and 29 UK-wide – some UK-wide sources also include Scotland editions) with essentially no gaps in coverage. Note, however, that some of these sources are the online versions of the newspapers’ print editions^13^ or their Sunday editions, meaning the actual number of newspapers included in the sample (as opposed to the number of sources) is 37. As for Italy, since by 2017 all but two metrics had been launched, I selected again 2017 as the start year of my search. The final sample included 58 sources with almost no gaps in coverage. Note again that some sources are the online versions of the newspapers’ print editions, meaning the actual number of newspapers included in the sample is 33.

Due to the high volume of articles yielded, I could not read each of them and exclude irrelevant ones manually. Thus, I had to find a search string that would enable Factiva to limit the number of potentially irrelevant articles returned.^14^ After years of revisions, I elaborated the following search string template (for a list of all strings I used and for further notes see Appendices 4 and 5): *metric’s name or official alternative metric’s name if available or unofficial alternative metric’s name if commonly used or [(main word(s) in metric’s name or metric’s acronym if commonly used) near15 (index or report or reports or survey or surveys or list or lists or ranking or rankings or league table or league tables)] near15 (developer’s name or alternative developer’s name if available or developer’s acronym if commonly used)*

First, we have the full metric’s name, followed by any official and unofficial alternative names. The remainder of the string was meant to return all articles in which metrics were mentioned without explicit reference to their name (as in e.g. “a recent *report* by the *United Nations* found *happiness* to…”). The *main word(s) in metric’s name* is e.g. happiness in the case of the WHR. This had to be within 15 words of at least one among a list of keywords, in either order. I took some of these keywords from the names of the metrics themselves (index, report, survey). To these, I added a few synonyms (ranking, list, league table); occasionally, the word indicator; and the plural version of all the above words. It is possible that some journalists will have never used any of these keywords. However, not adding them would have returned too many irrelevant articles. Finally, to limit the number of irrelevant results returned all these queries had to be found within again 15 words of the developer’s name, any alternative name or their acronym, if commonly used (e.g. UN).

As for metrics’ acronyms, I only searched for those of the HDI and of the BES as it is unlikely that journalists will have cited e.g. the IWI without explaining what this stands for, in addition to that of the PIQ as this is meant to be cited this way (see Appendix 5). The reason why I did not search for metrics’ acronyms by themselves is because each of them can stand for many different things. By ensuring they had to be mentioned within 15 words of the above keywords and the developer’s name, and by also including, when needed, an additional query to exclude articles in which irrelevant spelled-out forms were used,^15^ I limited considerably the number of potentially irrelevant articles returned.

Having said that, I used the above string in all but three cases. This is because two metrics lack a single official developer (GPI, ISEW) and, in the case of the GNH, there is no other way to refer to it without mentioning its full name. In these cases, I just searched for them by their full names. Moreover, for every query I also searched for any alternative spelled-out versions (e.g. *Indice dello/di sviluppo umano*) or any alternative spellings (e.g. Organization/Organisation) of the queries and words I used and, in the case of Italy, I ran searches both in English (when the metric’s original name was in English) and in Italian, contrarily to Morse (2015).

Finally, I also added GDP to the list of metrics that I searched for, for comparison similarly to Morse (2018). To be sure, GDP can be mentioned for many reasons which may not always be related to well-being. Therefore, in addition to *GDP* and *Gross Domestic Product* I also searched for the queries *economic growth* (which identifies a specific trend of GDP which is deemed necessary for well-being, both by actors inside and outside the well-being policy circle – see Hayden & Wilson, 2016, 2017, 2018a, b; Seaford, 2013; Thiry et al., 2013) and *recession*.^16^ Note that I could not use a string such as *(economic growth OR economy near15 grow*)* for it would have returned too many irrelevant results. That is why the queries I searched for were in this case the simplest ones and means that while the number of articles returned that mentioned any of the well-being metrics in the two countries’ samples is as accurate as possible, that in which the above queries were so is by far an underestimation.

Given the large number of articles returned, I could not read every article and delete duplicates manually. I therefore had to rely on Factiva’s duplicate identifier which allows to automatically exclude articles that are virtually identical. Additionally, due to the way Factiva works it was impossible for me to exclude duplicates in the case of articles mentioning GDP and related queries. When comparing the number of articles in which these were mentioned with that in which well-being metrics were so, I therefore included duplicates in the latter as well.

### TVEyes

On TVEyes, one can search transcripts of anything that was aired in the previous six months, plus the month one is running the search in. This means that as soon as the month in which TVEyes is being used ends, all transcripts older than six months get deleted. Source-wise, no gaps in coverage were recorded. Nonetheless, several sources were added between 2020 and 2021. These sources were excluded but this made no difference since almost no references were made on them anyway. In the end, 32 radio stations (16 UK-wide + 16 Scottish) and 13 TV channels (9 UK-wide + 4 Scottish) were analysed for Scotland, and 16 radio stations and 21 TV channels for Italy (for a full list, see Appendix 3).

The metrics that I searched for were the same ones I searched for on Factiva.^17^ Contrarily to Factiva, however, TVEyes seems incapable of handling too complicated and too many queries simultaneously. Additionally, until mid-2021 TVEyes did not allow the use of the *near* operator. For these reasons, I could not use a single search string. Instead, I had to search for queries separately (for a full list, see Appendices 4 and 5). In short, I searched for metrics again by their full name and any official or unofficial alternative names, as well as any acronyms if commonly used, both in English and Italian, using alternative spellings of the words I used and alternative spelled-out versions of the metrics’ names. I also searched for the main word(s) in any metric’s name alongside the name of their developer (or their acronym if commonly used), though without a list of keywords this time (except in three cases, see Appendix 5). However, in a few cases the use of this string was either not possible or not needed (see Appendices 4 and 5).

Contrarily to Factiva, TVEyes does not automatically identify duplicates which is a problem given the number of sister/regional stations and channels in Scotland and the UK. This means that if a reference to the WHR is made, for instance, on *Rai Radio 1* which is broadcast all over Italy, TVEyes will return one mention, whereas if the same reference is made on stations belonging to the British Broadcasting Corporation (BBC) such as *BBC Radio Shetland, BBC Radio Orkney, BBC Radio Scotland* and *BBC Radio Highlands & Islands*, it will return four instead. To avoid including duplicates, I thus had to remove them manually.

For a reference to be labelled as duplicate, three criteria had to be met. The first one was time. Indeed, it was very common to find the same show broadcast with a few seconds’ or minutes’ difference on multiple sources, though hardly with more than 5 min’ difference, which is why for a reference to be labelled as duplicate this needed, first, to have been made on the same day and at a very similar time as another one (± 5 min from each other). Second, for a reference to be considered the duplicate of another, their transcripts had to be either the same or very similar. Very similar and not just the same because sometimes the same show would be transcribed differently due to it being broadcast with a few variations on different stations belonging to the same network, especially when different news presenters reported the same news bulletin with different accents or changing the local news part or the weather forecasts. Third and last, references which met the conditions above had to appear either on sources belonging to the same network (e.g. *BBC Radio 1* and *BBC Radio 2*) or on sources belonging to the same company (e.g. *Kerrang!* and *Absolute Radio*). Indeed, many among the latter do not have a news desk and, even if they do, they often broadcast news bulletins from major news providers such as *Sky News*, which is why their transcripts would often look the same.

Finally, it is worth noting that contrarily to Factiva TVEyes shows the total number of mentions. However, if the query one is searching for was mentioned more than once within 30 s, TVEyes will only return one result. This means that not all actual mentions were counted, though from what I could see it was hardly the case for a metric to be mentioned twice or more in a 30-s timeframe.

## Appendix 2

### List of Newspapers

**Table:**

Scotland	Italy
Airdrie and Coatbridge Advertiser	Avvenire
Ayrshire Post	Avvenire Online
Blairgowrie Advertiser	Citynews Italy
Daily Record	Corriere Adriatico
dailyrecord.co.uk	Corriere Adriatico Online
Dumfries and Galloway Standard	Corriere del Sud
East Kilbride News	Corriere delle Alpi
Evening Express	Corriere della Sera^18^
Evening Times	Espresso.it^19^
Hamilton Advertiser	Gazzetta di Mantova
Holyrood	Gazzetta di Mantova Online
Kilmarnock Standard	Gazzetta di Parma Online News
Lennox Herald	Il Fatto Quotidiano
Paisley Daily Express	Il Fatto Quotidiano Online
Perthshire Advertiser	Il Gazzettino
Stirling Observer	Il Gazzettino Online
Strathearn Herald	Il Giornale
Sunday Herald	Il Giorno
Sunday Mail	Il Mattino
Sunday Sun	Il Mattino Online
The Galloway News	Il Mattino di Padova
The Herald	Il Messaggero
The Irvine Herald	Il Messaggero Online
The Press and Journal	Il Nuovo Quotidiano di Puglia Online
thescottishsun.co.uk	Il Piccolo
West Lothian Courier	Il Piccolo Online
Wishaw Press	Il Resto del Carlino
**UK-wide**	Il Secolo XIX Online
Daily Mail*	Il Sole 24 ORE Digital Replica of Print Edition
Daily Star Sunday	Il Sole 24 ORE – Online
Daily Star*	ItaliaOggi
dailystar.co.uk	ItaliaOggi7
express.co.uk	La Gazzetta dello Sport
Financial Times	La Nazione
Financial Times (FT.Com)^20^	La Nuova di Venezia e Mestre
i	La Provincia Pavese
Independent Online^21^	La Provincia Pavese Online
Mail Online	La Repubblica
Metro*	La Repubblica.it
Mirror.co.uk	La Repubblica Bari
Sunday Express*	La Repubblica Bologna
Sunday People*	La Repubblica Firenze
sundaytimes.co.uk	La Repubblica Genova
The Daily Express*	La Repubblica Milano
The Daily Mirror*	La Repubblica Napoli
The Daily Telegraph*	La Repubblica Palermo
The Guardian	La Repubblica Roma
The Independent	La Repubblica Torino
The Mail on Sunday	La Sentinella del Canavese
The Observer	La Sentinella del Canavese Online
The Sun*	La Stampa^22^
The Sunday Telegraph*	La Stampa Online^23^
The Sunday Times*	La Tribuna di Treviso
The Telegraph Online	Leggo
The Times*	L'Espresso
thesun.co.uk	Linkiesta.it
thetimes.co.uk	Messaggero Veneto
	Messaggero Veneto Online
**Tot: 56 (27 + 29)**	**Tot: 58**

*****includes Scotland edition.

### Notes

To collect information about all the Scottish, UK-wide and Italian sources covered by Factiva I had to click on every single source and take note of the type of coverage provided and the date this started, ceased or was suspended, as well as the language and the country of origin of each publication (this took me four months to complete). Lists of sources available can be found by opening the Search Builder, clicking on Select Source Category, By Region, and then on the country of choice. As regards the UK, sources can only be filtered by Sub-Region. This means that I could get a list of all Scottish sources, but not of UK-wide ones. However, sources without Sub-Region codes, as in the case of UK-wide titles, and particularly UK-wide newspapers, can be found under By Type, By Newspaper, and by then selecting the Newspapers: UK filter (at the time of writing, April 2022, the above process cannot be followed as such filter does not include UK-wide newspapers only anymore but all newspapers that circulate within the UK). For a detailed list of all supplements and local editions covered, please refer to the information provided by Factiva under the “i” button next to sources which can be retrieved either by following the above process or by searching for every source manually in the Search Builder.

## Appendix 3

### List of Radio Stations and TV Channels

**Table:**

Scotland	Italy
**Radio (owner)**	**Radio (owner)**
BBC Radio Scotland^24^	GR Parlamento (Rai)
Central 103 FM (New Wave Media/John Quinn)	ISORADIO (Rai)
CFM Carlisle (Bauer)	R101 (RadioMediaset)
Clyde 1 (Bauer)	Radio 105 (RadioMediaset)
Forth 1 (Bauer)	Radio 24 (Gruppo 24 ORE/Confindustria)
Forth 2 (Bauer)	Radio Capital (GEDI)
Heart Scotland East (Global)	Radio Deejay (GEDI)
Heart Scotland West (Global)	Radio Popolare (ERREPI)
Kingdom FM (DC Thomson)*	Radio Radicale (Centro di Produzione S.p.A./Associazione Marco Pannella)
Moray Firth (Bauer)	Radio Rai 1 (Rai)
Northsound 1 (Bauer)	Radio Rai 2 (Rai)
Original 106 FM (DC Thomson)**	Radio Rai 3 (Rai)
Radio Borders (Bauer)	Rai Sender Bozen (Rai)
Tay FM Dundee (Bauer)	RDS (Radio Dimensione Suono S.p.A.)
Wave 102/Pure Radio^25^ (DC Thomson)	RMC (RadioMediaset)
West Sound (Bauer)	RTL 102.5 (RTL 102.5 Hit Radio S.r.l.)
**TV (owner)**	**TV (owner)**
BBC Scotland (BBC)	Antenna 3 (Mediapason)
BBC 1 Scotland (BBC)	Canale 5 (Mediaset)
ITV1 Border (ITV)	Class CNBC (Class Editori)
STV (part of the ITV network)	Italia 1 (Mediaset)
**UK-wide**	La7 (Cairo Communication S.p.A.)
**Radio (owner)**	Milanow (Mediapason)
Absolute Radio (Bauer)	Odeon24 (Gruppo Striscione)
Capital XTRA (Global)	Rai 1 (Rai)
BBC Radio 1 (BBC)	Rai 2 (Rai)
BBC Radio 2 (BBC)	Rai 3 (Rai)
BBC Radio 1Xtra (BBC)	Rai 5 (Rai)
BBC Radio 3 (BBC)	Rai News (Rai)
BBC Radio 4 (BBC)	Rai Sport (Rai)
BBC Radio 5 Live (BBC)	Rete Quattro (Mediaset)
BBC Radio 6 Music (BBC)	Sky News TG 24 (Sky Italia)
Kerrang! (Bauer)	Telecity/7GOLD (7 Gold S.r.l.)
LBC (Global)	Telelombardia (Mediapason)
LBC News (Global)	Telenova (Multimedia San Paolo S.r.l.)
Share Radio (Share Premium Ltd)	TGcom24 (Mediaset)
talkRADIO (Wireless)	TG Norba 24 (Telenorba S.p.A.)
talkSPORT (Wireless)	TV8 (Sky Italia)
XFM (Global)	
**TV (owner)**	
5STAR (ViacomCBS)	
BBC Four (BBC)	
BBC News 24 (BBC)	
BBC Parliament (BBC)	
Channel 4 (Channel Four Television Corporation)	
FIVE (ViacomCBS)	
Sky News (Sky)	
Sky Sports F1 (Sky)	
Sky Sports News (Sky)	
**Tot: 45 (16 + 16) + (9 + 4)**	**Tot: 37 (16 + 21)**

*Acquired by DC Thomson in March 2019.

**Acquired by DC Thomson in March 2019.

### Notes

I attributed references made on Kingdom FM and Original 106 FM after the above dates to DC Thomson, and those made before to their previous owner, labelling duplicates accordingly.

## Appendix 4

### List of Queries Searched for – Scotland

#### Notes

Factiva is not case-sensitive. TVEyes is only case-sensitive when it comes to the use of operators such as AND or OR.

Double quotation marks were used to locate exact results. Except in one case (see notes on the BLI), they were not however used on Factiva since this already locates exact results.

As far as TVEyes is concerned, it is worth noting that in some cases it was not possible to use the “*main word in metric’s name” AND (“developer’s name” OR “acronym if commonly used”)* string. When a common query like *quality of life* was associated with a less common one like *Monocle*, this would usually return 0–50 results which would enable me to look at them and manually delete any irrelevant mentions (it turned out all of them were). When none of the queries were common (as in *vibrant economy* and *Grant Thornton*), things would be even easier as TVEyes would usually return no results at all. However, when both queries were popular, or when one was the result of a mistranscription, the number of results returned would be too high for me to even skim read their transcripts which is why the above string could not be used. Searching for *“Human Development” AND (“UN” OR “United Nations”)*, for instance, would have returned all segments in which speakers mentioned the UN and its work on human development, or quoted someone from the UN talking about human development, or in which words such as ‘un-necessary’ and ‘human development’ appeared together, even though in all such cases no one referenced to the HDI.

In the case of TVEyes I had to search for queries separately (each individual query is presented below on a different line or through indentation). This means that after searching for e.g. a metric by its full name, I often had to add *NOT “metric’s full name”* at the end of other queries to avoid including duplicates because searching for e.g. “*happiness” AND (“UN” OR “United Nations”)* returns also mentions of the WHR which will have already been returned for *World Happiness Report*.^26^

**Table:**

Metric	Factiva (Newspapers)	TVEyes (Radio and TV)
Bank of Scotland QoLS	**Bank of Scotland Quality of Life Survey or Halifax Quality of Life Survey or [Quality of Life near15 (index or report or reports or survey or surveys or list or lists or ranking or rankings or league table or league tables)] near15 (Bank of Scotland or Halifax)**	**“Bank of Scotland Quality of Life Survey”** **(“Bank of Scotland” AND “Quality of Life”) NOT “Bank of Scotland Quality of Life Survey”** **“Halifax Quality of Life Survey”** **(“Halifax” AND “Quality of Life”) NOT “Halifax Quality of Life Survey”**
	The indicators in the Bank of Scotland and Halifax surveys are the same, the only difference is that the latter is measured for the UK whereas the former is measured for Scotland only and therefore branded as a Bank of Scotland output (both Halifax and Bank of Scotland are part of the Lloyds Banking Group). The Halifax QoLS is therefore relevant to a Scottish audience (whereas the Bank of Scotland QoLS will not be relevant to a Welsh audience), which is why both names were searched for	Contrarily to Italy (see Appendix 5), in the case of Scotland I did not add a list of keywords such as *report* after e.g. *(“Halifax” AND “Quality of Life”)*. This is because contrarily to e.g. ISTAT or *Sole 24 ORE*, Halifax is not used as widely (since Halifax does not issue official statistics or own TV channels or radio stations), meaning the number of results returned was always small and this did not require the addition of other words. Note that I could not search for e.g. *(“Halifax” AND “Quality of Life”) NOT (“Halifax Quality of Life Survey” OR “Bank of Scotland Quality of Life Survey”)*, so I checked manually whether Halifax references had already been counted under Bank of Scotland and vice versa
	There was no need to add the Saxon genitive to the metric’s name as in *Bank of Scotland’s Quality of Life Survey* since items in which this expression was used were captured by the second half of the string in the case of Factiva and the *(“main word in metric’s name” AND “developer’s name”)* string in the case of TVEyes	
BCI	**Basic Capabilities Index or Basic Capabilities Report or [Basic Capabilities near15 (index or report or reports or survey or surveys or list or lists or ranking or rankings or league table or league tables)] near15 Social Watch**	**“Basic Capabilities Index”** **“Basic Capabilities Report”** **"Basic Capabilities” AND “Social Watch”**
BLI	**Better Life Index or How's Life Report or How is Life Report or [(Better Life or How is Life or How's Life) near15 (index or report or reports or survey or surveys or list or lists or ranking or rankings or league table or league tables)] near15 (OECD or “Organi?ation for Economic Co-operation and Development” or “Organi?ation for Economic Cooperation and Development”)**	** “Better Life Index”** **“How is Life Report”** **“How’s Life Report”** **“Better Life” OR “How’s Life” OR “How is Life”) AND (“OECD” or “Organisation for Economic Co-operationand Development”)**
	The ? sign is used to yield articles in which both the word organisation and organization have been used. Double quotation marks were in this case used since the full name of the OECD contains *and* in it which Factiva therefore interprets as the *and* operator	Both with and without the hyphen and with *Organization* in place of *Organisation*. Note that in the case of TVEyes coverage of the second and third queries starts from June 2019. However, this is unlikely to have affected findings since both queries never returned any results since (not to mention that no How is Life Report was published in 2018)
EURSPI	Given that the EURSPI contains the words *Social Progress Index* in its name, I just read the items in which mentions of the SPI were found to see which ones were about the SPI and which ones about the EURSPI instead	
GCI	**Good Country Index or Good Country Report or [Good Country near15 (index or report or reports or survey or surveys or list or lists or ranking or rankings or league table or league tables)] near15 Simon Anholt**	**“Good Country Index”** **“Good Country Report”** **“Good Country” AND “Simon Anholt”**
GGCI	**Good Growth for Cities Index or Good Growth for Cities Report or [Good Growth near15 (index or report or reports or survey or surveys or list or lists or ranking or rankings or league table or league tables)] near15 (PwC or PricewaterhouseCoopers)**	**“Good Growth for Cities Index”** **“Good Growth for Cities Report”** **“Good Growth” AND (“PwC” OR “PricewaterhouseCoopers”)**
		Note that coverage of the first two queries starts from June 2019. They never returned any results since, and the GGCI as the analysis of newspapers also showed was among the least mentioned metrics in the sample, hence this small gap in coverage will have had little to no effect
GIP	**Glasgow Indicators Project or [Understanding Glasgow near15 (index or indicator or indicators or report or reports or survey or surveys or list or lists or ranking or rankings or league table or league tables)] near15 (Glasgow Centre for Population Health or Glasgow Center for Population Health or GCPH)**	**“Understanding Glasgow”** **“Glasgow Indicators Project”**
	The words *indicator* and *indicators* were added given that the GIP is a dashboard of indicators, hence journalists may have used them instead of e.g. report. *Understanding Glasgow* was not searched for individually because it is an expression that could be used for other purposes as well	There was no need to add other queries because the ones above returned a handful of results each, in fact often none at all, hence I could just go through each of them manually
GNH	**Gross National Happiness**	**“Gross National Happiness”**
GPI	**Genuine Progress Indicator**	**“Genuine Progress Indicator”**
HDI	**Human Development Index or Human Development Report or [(Human Development or HDI) near15 (index or report or reports or survey or surveys or list or lists or ranking or rankings or league table or league tables)] near15 (UN or United Nations)**	**“Human Development Index”** **“Human Development Report”** **“HDI”**
		As I explained above, I could not add the query “*Human Development” AND (“UN” OR “United Nations”)* given the popularity of the words in question. Note that for some reasons I cannot retrieve data for the period of November 2018-April 2019. While there is therefore a small gap in coverage, the remaining 85% of coverage, which includes the releases of the 2019 and 2020 HDI (no HDI was issued in 2021), is unaffected
HKI	**Humankind Index or Humankind Report or Human Kind Index or Human Kind Report or [(Humankind or Human Kind) near15 (index or report or reports or survey or surveys or list or lists or ranking or rankings or league table or league tables)] near15 Oxfam**	**“Humankind Index”** **“Human kind Index”** **“Humankind Report”** **“Human kind Report”**
	The query *human kind* was added because contrarily to all other metrics the HKI contains a word in its name (humankind) which may have been written/transcribed incorrectly as two separate words (as indeed I found to be the case)	
		Due to *Humankind* and *Oxfam* being common words, I could not add the query “*Humankind” AND “Oxfam”* (I did search for it anyway unofficially to make sure I was not missing relevant mentions, but I never found any)
HPI	**Happy Planet Index or Happy Planet Report or [Happy Planet near15 (index or report or reports or survey or surveys or list or lists or ranking or rankings or league table or league tables)] near15 (NEF or New Economics Foundation or WEAll or Wellbeing Economy Alliance)**	**“Happy Planet Index”** **“Happy Planet Report”** **“Happy Planet” AND (“NEF” OR “New Economics Foundation”)** **“Happy Planet” AND (“WEAll” OR “Wellbeing Economy Alliance”)**
	The last two queries in the case of Factiva and the fourth queries in the case of TVEyes were added in 2022 since the 5^th^ iteration of the HPI, published in October 2021, was supported by WEAll and not NEF (having subscribed to TVEyes for the last time in January 2022, in the case of TVEyes coverage of this last query is limited from July to December 2021)	
ISEW	**Index of Sustainable Economic Welfare**	**“Index of Sustainable Economic Welfare”**
IWI	**Inclusive Wealth Index or Inclusive Wealth Report or [Inclusive Wealth near15 (index or report or reports or survey or surveys or list or lists or ranking or rankings or league table or league tables)] near15 (UN or United Nations)**	**“Inclusive Wealth Index”** **“Inclusive Wealth Report”** **“Inclusive Wealth” AND (“UN” OR “United Nations”)**
LPI	**Legatum Prosperity Index or Legatum Prosperity Report or [Prosperity near15 (index or report or reports or survey or surveys or list or lists or ranking or rankings or league table or league tables)] near15 Legatum**	**“Prosperity Index”** **“Prosperity Report”**
	There was no need to add the Saxon genitive to the metric’s name as in *Legatum’s Prosperity Index* since articles in which such expression was used were captured by the second half of the string	I did not add *Legatum* since, being it a Latin word, there is a high chance that TVEyes will not transcribe it correctly. Instead, I used a simpler string and excluded any irrelevant results manually. I also did not search for “*Prosperity” AND “Legatum”* because of the commonality of the word prosperity and the fact that, as I found, people from the Legatum Institute are frequently interviewed and often use the word prosperity when talking to journalists about prosperity in general without necessarily referring to the LPI
Monocle QoLS	**Monocle Quality of Life Survey or [Quality of Life near15 (index or report or reports or survey or surveys or list or lists or ranking or rankings or league table or league tables)] near15 Monocle**	**“Monocle Quality of Life Survey”** **“Quality of Life” AND “Monocle”**
	There was no need to add the Saxon genitive to the metric’s name as in *Monocle’s Quality of Life Survey* since items in which such expression was used were captured by the second half of the string in the case of Factiva and the *(“main word in metric’s name” AND “developer’s name”)* strings in the case of TVEyes	
NPF	**(National Performance Framework or Scotland Performs) near15 (Scottish Government or wellbeing or well-being or progress or website)**	**“National Performance Framework”** **“NPF”** **“Scotland Performs”**
	Searching for *National Performance Framework* only would have returned too many irrelevant results as different National Performance Frameworks exist in the world. The same applies to Scotland Performs, which if searched for alone returns articles in which journalists talk about how Scotland performs compared to e.g. England (once again, Factiva is not case-sensitive). This is the reason why I added a few keywords such as *website* and *progress* since Scotland Performs used to be the website where one could find information about progress made by the Scottish Government in achieving the objectives of the NPF	Contrarily to Factiva, I added *NPF* because, given the smaller number of items TVEyes searches, fewer results were returned so I could check manually whether they were about the NPF or not. Also, it is more likely that a policymaker will have used the acronym NPF in a radio conversation in which they already explained what the NPF stands for rather than in a newspaper article in which there is less space for doing that. As already explained above, every time I searched for metrics by their acronyms I manually went through every item returned and double checked if the mentions identified were actually about the metrics I was interested in. Note that this applies to the query *Scotland Performs* as well, meaning I manually excluded all references which were about how e.g. “Scotland performs compared to…”
RMHI	**Royal Mail Happiness Index or Royal Mail Happiness Report or [Happiness near15 (index or report or reports or survey or surveys or list or lists or ranking or rankings or league table or league tables)] near15 Royal Mail**	**“Royal Mail Happiness Index”** **“Royal Mail UK Happiness Index”** **“Happiness Index” AND “Royal Mail”**
		I had to add *Index* to the third query because given the commonality of *happiness* and *Royal Mail* searching for “*Happiness” AND “Royal Mail”* would have returned hundreds of irrelevant results
	There was no need to add the Saxon genitive to the metric’s name as in *Royal Mail’s Happiness Index* since items in which such expression was used were captured by the second half of the string in the case of Factiva and the *(“main word in metric’s name” AND “developer’s name”)* strings in the case of TVEyes	
SPI	**Social Progress Index or Social Progress Report or [Social Progress near15 (index or report or reports or survey or surveys or list or lists or ranking or rankings or league table or league tables)] near15 Social Progress Imperative**	**“Social Progress Index”** **“Social Progress Report”** **“Social Progress” AND “Social Progress Imperative”**
uSwitch QoLI	**uSwitch Quality of Life Index or uSwitch’s Quality of Life Index or [Quality of Life near15 (index or report or reports or survey or surveys or list or lists or ranking or rankings or league table or league tables)] near15 uSwitch**	**“uSwitch Quality of Life Index”** **“Quality of Life” AND “uSwitch”**
	There was no need to add the Saxon genitive to the metric’s name as in *uSwitch’s Quality of Life Index* since items in which such expression was used were captured by the second half of the string in the case of Factiva and the *(“main word in metric’s name” AND “developer’s name”)* strings in the case of TVEyes	
VEI	**Vibrant Economy Index or Vibrant Economy Report or [Vibrant Economy near15 (index or report or reports or survey or surveys or list or lists or ranking or rankings or league table or league tables)] near15 Grant Thornton**	**“Vibrant Economy Index”** **“Vibrant Economy Report”** **“Vibrant Economy” AND “Grant Thornton”**
WHR	**World Happiness Report or World Happiness Index or [Happiness near15 (index or report or reports or survey or surveys or list or lists or ranking or rankings or league table or league tables)] near15 (UN or United Nations)**	**“World Happiness Report”** **“World Happiness Index”** **(“Happiness Index” AND “UN”) NOT (“World Happiness Index” OR “World Happiness Report”)** **(“Happiness Index” AND “United Nations”) NOT (“World Happiness Index” OR “World Happiness Report”)** **(“Happiness Report” AND “UN”) NOT (“World Happiness Index” OR “World Happiness Report”)** **(“Happiness Report” AND “United Nations”) NOT (“World Happiness Index” OR “World Happiness Report”)**
	World Happiness Index is the only unofficial alternative name I searched for, which as I expected and as I found is often misused as a synonym for the WHR, despite the former being another metric	
		Given that the first two queries yielded hundreds of results, I had to add the *NOT* part to the remaining strings to avoid including the same references twice
WWF LPI	**Living Planet Index or Living Planet Report or [Living Planet near15 (index or report or reports or survey or surveys or list or lists or ranking or rankings or league table or league tables)] near15 (WWF or World Wide Fund or Zoological Society of London or ZSL)**	**“Living Planet Index”** **“Living Planet Report”**
		Given that the WWF owns a Living Planet Centre and that *Living Planet* and *WWF* are therefore both common queries, as well as the fact that the word WWF could be returned as a mistranscription of other words in the case of TVEyes, I could not search “*Living Planet” AND “WWF”*

## Appendix 5

### List of Queries Searched for – Italy

#### Notes

See notes above in Appendix 4.

In the case of TVEyes I could not use elaborated strings with too many queries. This means I could not use a list of keywords like the one I searched for on Factiva but instead had to search for less advanced strings separately and then delete any irrelevancies manually. However, in three cases (those of the BES and of the ItaliaOggi and Sole 24 ORE QoLRs) I had to add some keywords because otherwise too many irrelevant results would have been returned. Once again, I could not add too many queries at the same time, hence I could only use some of the keywords that I searched for on Factiva.

**Table:**

Metric	Factiva (Newspapers)	TVEyes (Radio and TV)
ABR	**Classifica del ben vivere or classifica del benvivere or classifica sul ben vivere or classifica sul benvivere or classifica benvivere or classifica ben vivere or [(ben vivere or benvivere) near15 (indice or rapporto or rapporti or classifica or classifiche or graduatoria or graduatorie or ranking or dossier or indagine or indagini or mappa or mappe)] near15 Avvenire**	**(“ben vivere” OR “benvivere”) AND “Avvenire”**
	Contrarily to the search string I used in the case of other quality of life rankings (such as the *Sole 24 ORE*’s), I added the first six queries because in a small number of articles the ABR was mentioned by *Avvenire* without any references to the name of the newspaper. Being the ranking published by it, it makes sense that they will have not always mentioned their name when talking about their own ranking. This again applies only to this ranking and not to *Sole 24 ORE*’s and *ItaliaOggi*’s. This is because the latter have the same name (so searching for *Classifica della qualità della vita* returns articles in which both the Sole 24 ORE and the ItaliaOggi QoLRs were mentioned) and because both metrics also include a much more common expression (quality of life), whereas in the case of the ABR *ben vivere* and *benvivere* are very uncommon so any articles mentioning the *classifica del benvivere* will almost certainly be about the ABR. Note also that *mappa* and *mappe* (tr. map, maps) were added because both terms are occasionally used by journalists to refer to quality of life rankings specifically	Contrarily to the search string I used in the case of other quality of life rankings (such as the *Sole 24 ORE*’s), there was no need to add a list of keywords such as *rapporto* or *graduatoria* because again the words *benvivere* and *ben vivere* are very uncommon, so using this string returned very few results which enabled me to double check them manually
BCI	**Basic Capabilities Index or Basic Capabilities Report or indice di capacità di base or indice delle capacità di base or rapporto delle capacità di base or rapporto sulle capacità di base or [(Basic Capabilities or capacità di base) near15 (index or report or indice or rapporto or rapporti or classifica or classifiche or graduatoria or graduatorie or ranking or dossier or indagine or indagini)] near15 Social Watch**	**“Basic Capabilities Index”** **“Basic Capabilities Report”** **“Indice di capacità di base”** **“Indice delle capacità di base”** **“Rapporto delle capacità di base”** **“Rapporto sulle capacità di base”** **“Capacità di base”**
		Since the BCI was never mentioned, when searching for the last query there was no need to add *AND “Social Watch”* as the query in question never returned any results
BES	**Benessere equo e sostenibile or [(benessere or BES) near15 (indice or indici or rapporto or rapporti or classifica or classifiche or graduatoria or graduatorie or ranking or dossier or indagine or indagini or indicatore or indicatori) NOT bisogni educativi speciali] near15 (ISTAT or Istituto Nazionale di Statistica)**	**“Benessere equo e sostenibile”** **(“BES” NOT “benessere equo e sostenibile”) AND (“indicatore” OR “indicatori” OR “indice” OR “indici” OR “rapporto” OR “rapporti” OR “report” OR “indagine”)**
	Like in the case of the GIP (see Appendix 4), I added the words *indicatore* and *indicatori* because several BES *indicators* were included in the DEF. I added the query *indici* because given the above journalists may have well used indices as a synonym for indicators or to refer to the different composite indices included in the BES being this a hybrid. I did not search for *benessere and (ISTAT or Istituto Nazionale di Statistica)* because this would have yielded hundreds of irrelevant results given the popularity of the first two terms particularly	
		Searching for *BES* without any additional keywords will have returned thousands of irrelevant results since it could be a mistranscription of other words (particularly of the word ‘beh’, which means well as in ‘well, I think that…’ and which is frequently used in everyday language). Searching for *BES* using the above keywords returned irrelevant results, too – about 98% – but helped to reduce them significantly
BLI	**Better Life Index or How's Life Report or How is Life Report or indice di vita migliore or indice della vita migliore or indice di una vita migliore or indice di migliore vita or indice della migliore vita or indice di una migliore vita or rapporto come va la vita or rapporto la vita come va or rapporto su come va la vita or rapporto sulla vita come va or rapporto di come va la vita or rapporto della vita come va or [(Better Life or How is Life or How's Life or migliore vita or vita migliore or come va la vita or la vita come va) near15 (index or report or indice or rapporto or rapporti or classifica or classifiche or graduatoria or graduatorie or ranking or dossier or indagine or indagini)] near15 (“Organi?ation for Economic Co-operation and Development” or “Organi?ation for Economic Cooperation and Development” or Organizzazione per la cooperazione e lo sviluppo economico or Organizzazione per la co-operazione e lo sviluppo economico or OECD or OCSE)**	**“Better Life Index”** **“How’s Life Report”** **“How is Life Report”** **(“Better Life” OR “How’s Life” OR “How is Life”) AND (“OECD” OR “OCSE” OR “Organizzazione per lo sviluppo e la co-operazione economica”)** **“Indice di vita migliore”** **“Indice di migliore vita”** **“Indice della vita migliore”** **“Indice della migliore vita”** **“Indice di una vita migliore”** **“Indice di una migliore vita”** **“Come va la vita” OR “migliore vita”) AND (“OECD” OR “OCSE” OR “Organizzazione per la Co-operazione e lo Sviluppo Economico”** **(“Come va la vita” OR “migliore vita”) AND (“OECD” OR “OCSE” OR “Organizzazione per la Co-operazione e lo Sviluppo Economico”)**
	See notes above on the BLI in Appendix 4 regarding the use of double quotation marks	Both with and without the hyphen
EURSPI	See notes on the EURSPI in Appendix 4. In the case of Scotland, all variations of the EURSPI’s name always include the words ‘social progress’ (e.g. index of regional social progress, regional social progress index), hence there was no need to use additional queries. In the case of Italy, however, different possible translations of the EURSPI exist in which the words ‘progresso sociale’ may not always appear one after the other. For this reason, in addition to any references to the EURSPI found among references to the SPI, mentions returned using the following queries were also counted	
	**Indice del progresso regionale sociale or indice di progresso regionale sociale or rapporto del progresso regionale sociale or rapporto sul progresso sociale regionale or [progresso regionale sociale near15 (index or report indice or rapporto or rapporti or classifica or classifiche or graduatoria or graduatorie or ranking or dossier or indagine or indagini)] near15 (Social Progress Imperative or UE or Unione europea or European Union or EU)**	**“Indice del progresso regionale sociale”** **“Indice di progresso regionale sociale”** **“Rapporto del progresso regionale sociale”** **“Rapporto sul progresso sociale regionale”**
GNH	**Gross National Happiness or felicità interna lorda**	**“Gross National Happiness”** **“Felicità interna lorda”**
GCI	**Good Country Index or Good Country Report or indice del buon paese or indice del paese più buono or indice del paese buono or [(Good Country or paese buono) near15 (index or report or indice or rapporto or rapporti or classifica or classifiche or graduatoria or graduatorie or ranking or dossier or indagine or indagini)] near15 Simon Anholt**	**“Good Country Index”** **“Indice del buon paese”** **“Indice del paese più buono”** **“Indice del paese buono”** **(“Good Country” OR “paese buono” OR “buon paese”) AND “Simon Anholt”**
GPI	**Genuine Progress Indicator or indicatore del progresso genuino or indicatore del progresso reale or indicatore di progresso genuino or indicatore di progresso reale**	**“Genuine Progress Indicator”** **“Indicatore del progresso genuino”** **“Indicatore del progresso reale”** **“Indicatore di progresso genuino”** **“Indicatore di progresso reale”**
HDI	**Human Development Index or Human Development Report or indice di sviluppo umano or indice dello sviluppo umano or [(Human Development or sviluppo umano or HDI or ISU) near15 (index or report or indice or rapporto or rapporti or classifica or classifiche or graduatoria or graduatorie or ranking or dossier or indagine or indagini)] near15 (United Nations or Nazioni Unite or ONU)**	**“Indice di sviluppo umano”** **(“HDI” OR “ISU”) AND “Nazioni Unite”** **“Indice dello sviluppo umano”** **“Human Development Index”** **“Human Development Report”** **“Rapporto dello sviluppo umano”** **“Rapporto sullo sviluppo umano”**
	*UN* was not added because it means ‘the’ in Italian, hence it is a word that is used on an ordinary basis and since none of the tools is case-sensitive adding it would have returned thousands of irrelevant items. Note that I searched not only for the English acronym HDI but also for the Italian one ISU. However, irrelevancies and duplicates excluded, neither of them returned any results	
		The query *ONU* was excluded because it appears frequently in transcripts but only as a mistranscription of other words. Also note that the coverage of the fourth query starts from December 2019. However, this is unlikely to have affected findings since it only returned four references between December 2019 and December 2021, so the HDI does not seem to be reported much by its English name contrarily to what Morse (2015, 2016) assumed
HPI	**Happy Planet Index or Happy Planet Report or indice del pianeta felice or indice di un pianeta felice or rapporto del pianeta felice or rapporto di un pianeta felice or rapporto sul pianeta felice or [(Happy Planet or pianeta felice) near15 (index or report or indice or rapporto or rapporti or classifica or classifiche or graduatoria or graduatorie or ranking or dossier or indagine or indagini)] near15 (NEF or New Economics Foundation or WEAll or Wellbeing Economy Alliance)**	**“Happy Planet Index”** **“Happy Planet Report”** **“Indice del pianeta felice”** **(“Pianeta felice” OR “Happy Planet”) AND (“NEF” OR “New Economics Foundation”)** **“Pianeta felice” OR “Happy Planet”) AND (“WEAll” OR “Wellbeing Economy Alliance”)**
	See notes above on the HPI in Appendix 4	
ISEW	**Index of Sustainable Economic Welfare or indice di benessere economico sostenibile**	**“Index of Sustainable Economic Welfare”** **“Indice di benessere economico sostenibile”**
ItaliaOggi QoLR	**[qualità della vita near15 (indice or rapporto or rapporti or classifica or classifiche or graduatoria or graduatorie or ranking or dossier or indagine or indagini or mappa or mappe)] near15 (ItaliaOggi or Italia Oggi)**	**“Qualità della vita” AND “ItaliaOggi” AND (“graduatoria” OR “rapporto” OR “classifica” OR “dossier” OR “mappa” OR “ranking” OR “indagine” OR “report”)** **“Qualità della vita” AND “Italia Oggi” AND (“graduatoria” OR “rapporto OR “classifica” OR “dossier” OR “mappa” OR “ranking” OR “indagine” OR “report”)**
	Note that I did not only search for *ItaliaOggi* but also for *Italia Oggi* since a) these are used interchangeably; b) in the case of TVEyes, this might have transcribed them with or without spaces depending on how the speaker pronounced them; c) *ItaliaOggi* is composed by two common words, ‘Italia’ and ‘oggi’, which TVEyes again might have not recognised as the paper’s name but as to separated words; and d) to their regular usage which increased the likelihood of all points above. And I was right – in the case of TVEyes, using alternative spellings returned, duplicates and irrelevancies excluded, 116 additional results	
	See notes on the ABR regarding the addition of the queries *mappa* and *mappe*	As explained above, I had to add keywords like *rapporto* to limit the number of irrelevant mentions returned: searching for *“qualità della vita” AND* either “*ItaliaOggi”* or “*Sole 24 ORE”* would have yielded many irrelevant mentions due to the frequent usage and popularity of both the query *quality of life* and of the two newspapers’ names
IWI	**Inclusive Wealth Index or Inclusive Wealth Report or indice della ricchezza inclusiva or indice del benessere inclusivo or indice di ricchezza inclusiva or indice di benessere inclusivo or rapporto della ricchezza inclusiva or rapporto del benessere inclusivo or rapporto sul benessere inclusivo or rapporto sulla ricchezza inclusiva or [(Inclusive Wealth or ricchezza inclusiva or benessere inclusivo) near15 (index or report or indice or rapporto or rapporti or classifica or classifiche or graduatoria or graduatorie or ranking or dossier or indagine or indagini)] near15 (United Nations or ONU or Nazioni Unite)**	**“Inclusive Wealth Index”** **“Inclusive Wealth Report”** **“Indice di ricchezza inclusiva”** **“Indice della ricchezza inclusiva”** **“Indice di benessere inclusivo”** **“Indice del benessere inclusivo”** **“Inclusive Wealth” OR “ricchezza inclusiva” OR “benessere inclusivo”) AND “Nazioni Unite”** **“Ricchezza inclusiva”** **“Benessere inclusivo”**
	See notes above on the HDI regarding the exclusion of the query *UN*	See notes above on the HDI regarding the exclusion of the queries *UN* and *ONU.* See notes on the BLI regarding the last two queries
LPI	**Legatum Prosperity Index or Legatum Prosperity Report or indice della prosperità or indice di prosperità or rapporto della prosperità or rapporto sulla prosperità or [(prosperity or prosperità) near15 (index or report or indice or rapporto or rapporti or classifica or classifiche or graduatoria or graduatorie or ranking or dossier or indagine or indagini)] near15 Legatum**	**“Prosperity Index”** **“Indice della prosperità”** **“Indice di prosperità”** **“Prosperity Report”** **“Rapporto sulla prosperità”** **“Rapporto della prosperità”** **“Prosperità” AND “Legatum”**
		Note that the coverage of the fourth, fifth and sixth queries starts from June 2020. They returned no results since, and the LPI as the analysis of newspapers also showed was among the least mentioned metrics in the sample, hence this small gap in coverage will have had little to no effect at all. Additionally, the main names used to refer to the LPI, so the ones that will have been mentioned the most, were unaffected. Also note that contrarily to Scotland I was able to search for “*Prosperità” AND “Legatum”* because Legatum representatives are not interviewed in Italy as they are based in the UK, hence both queries are less likely to be found in the same transcript
PIQ	**Prodotto interno di qualità or (PIQ and Symbola)**	**“Prodotto interno di qualità”** **“PIQ” AND “Symbola”**
	I searched for *PIQ* as well because the aim of the PIQ was to be compared to GDP – the word PIQ itself sounds similar to the word PIL (GDP) – and it is thus likely that some will have cited its acronym only, even though this has not become as popular as e.g. HDI	
QUARS	**Indice di qualità dello sviluppo regionale or indice di qualità regionale dello sviluppo or indice della qualità dello sviluppo regionale or indice della qualità regionale dello sviluppo or rapporto della qualità regionale dello sviluppo or rapporto sulla qualità regionale dello sviluppo or rapporto sullo sviluppo di qualità regionale or rapporto della qualità dello sviluppo regionale or [(sviluppo regionale or QUARS) near15 (indice or rapporto or rapporti or classifica or classifiche or graduatoria or graduatorie or ranking or dossier or indagine or indagini)] near15 (Sbilanciamoci or Lunaria)**	**“QUARS”** **“Qualità dello sviluppo regionale”** **“Qualità regionale dello sviluppo”** **“Sviluppo regionale” AND (“Lunaria” OR “Sbilanciamoci”)**
Rome BES	**[Benessere equo e sostenibile near15 (Roma Capitale or Comune di Roma)] or [BES near15 (indice or indici or rapporto or rapporti or classifica or classifiche or graduatoria or graduatorie or ranking or dossier or indagine or indagini or indicatore or indicatori) NOT bisogni educativi speciali] near15 (Roma Capitale or Comune di Roma)**	**“Benessere equo e sostenibile” AND “Roma Capitale”** **“Benessere equo e sostenibile” AND “Comune di Roma”** **“BES” AND “Roma Capitale”** **“BES” AND “Comune di Roma”**
	Contrarily to the case of the BES, *benessere* was not added after *BES* because Rome’s Council has a directorate for Green Policies and Animal Wellbeing, so adding it will have returned irrelevant articles that mentioned either the directorate or the Council Assessor responsible for it	
Sole 24 ORE QoLR	**[qualità della vita near15 (indice or rapporto or rapporti or classifica or classifiche or graduatoria or graduatorie or ranking or dossier or indagine or indagini or mappa or mappe)] near15 (Sole 24 ORE or Sole24 ORE or Sole 24ORE or Sole24ORE or Soleventiquattrore or Sole ventiquattrore or Soleventiquattr'ore or Sole ventiquattr'ore)**	**“Qualità della vita” AND “Sole24Ore” AND (“graduatoria” OR “rapporto” OR “classifica” OR “dossier” OR “mappa” OR “ranking” OR “indagine”)** **“Qualità della vita” AND “Sole 24 Ore” AND (“graduatoria” OR “rapporto” OR “classifica” OR “dossier” OR “mappa” OR “ranking” OR “indagine”)**
	See notes on the ABR regarding the addition of the queries *mappa* and *mappe.* Note that in this and this case only the number of duplicates shown does not match the number of duplicates on the page if one were to count all duplicates manually. I informed Factiva’s Support Team which told me that they identified the cause of the problem but that the solution will require significant changes. At the time of writing (April 2022), the problem has not been fixed yet. As such, I counted all duplicates and the number of articles published by each source manually	See notes at the beginning and on the ItaliaOggi QoLR. There was no need to use alternative spelled-out versions of *Sole 24 ORE* because TVEyes recognises the paper’s name and as such it always transcribes it the correct way (I did try searching for e.g. *soleventiquattrore* but no results were ever returned)
	See notes on the ItaliaOggi QoLR	
SPI	**Social Progress Index or Social Progress Report or indice del progresso sociale or indice di progresso sociale or rapporto del progresso sociale or rapporto sul progresso sociale or [(Social Progress or progresso sociale) near15 (index or report or indice or rapporto or rapporti or classifica or classifiche or graduatoria or graduatorie or ranking or dossier or indagine or indagini)] near15 Social Progress Imperative**	**“Social Progress Index”** **“Indice del Progresso Sociale”** **“Indice di Progresso Sociale”** **“Social Progress Report”** **“Rapporto sul progresso sociale”** **“Rapporto del progresso sociale”** **“Progresso sociale” AND “Social Progress Imperative”**
		Note that the coverage of the last three queries starts from December 2019. However, as the analysis of newspapers also showed the SPI was among the least mentioned metrics in the sample, hence this small gap in coverage will have had little to no effect at all. Additionally, the main names used to refer to the SPI, so the ones that will have been mentioned the most, were unaffected
WHR	**World Happiness Report or World Happiness Index or indice mondiale di felicità or indice mondiale della felicità or indice della felicità del mondo or indice della felicità nel mondo or indice di felicità del mondo or indice di felicità nel mondo or rapporto mondiale della felicità or rapporto mondiale sulla felicità or rapporto della felicità del mondo or rapporto sulla felicità del mondo or rapporto della felicità nel mondo or rapporto sulla felicità nel mondo or [(happiness or felicità) near15 (index or report or indice or rapporto or rapporti or classifica or classifiche or graduatoria or graduatorie or ranking or dossier or indagine or indagini)] near15 (United Nations or Nazioni Unite or ONU)**	**“World Happiness Report”** **“World Happiness Index”** **“Rapporto mondiale sulla felicità” NOT (“World Happiness Report” OR “World Happiness Index”)** **“Rapporto mondiale della felicità” NOT (“World Happiness Report” OR “World Happiness Index”)** **“Felicità” AND “Nazioni Unite” NOT (“World Happiness Report” OR “World Happiness Index” OR “rapporto mondiale della felicità” OR “rapporto mondiale sulla felicità”)**
	See notes above on the HDI regarding the exclusion of the query *UN*	See notes above on the HDI regarding the exclusion of the queries *UN* and *ONU*. The last query was added to return articles in which the WHR may have been mentioned using expressions like ‘del mondo’ instead of ‘mondiale’, as well as alternative spelled-out versions of the WHR’s unofficial name (World Happiness Index). This query was not used for Scotland since the name of the WHR is not translated there
WWF LPI	**Living Planet Index or Living Planet Report or indice del pianeta vivente or indice di un pianeta vivente or rapporto del pianeta vivente or rapporto di un pianeta vivente or rapporto sul pianeta vivente or [(Living Planet or pianeta vivente) near15 (index or report or indice or rapporto or rapporti or classifica or classifiche or graduatoria or graduatorie or ranking or dossier or indagine or indagini)] near15 (WWF or World Wide Fund or Zoological Society of London or ZSL or Zoological Society di Londra or Società di zoologia di Londra or Società zoologica di Londra)**	**“Living Planet Index”** **“Living Planet Report”** **“Indice del pianeta vivente”** **“Indice di un pianeta vivente”**
		See notes in Appendix 4 on the WWF LPI regarding the exclusion of the string *(“main word in metric’s name” AND “developer’s name”)*

## Footnotes

1 In his 2018 study, Morse (2018, p. 436) did not even specify that, limiting to say that he chose metrics that ‘had been updated over a number of years’.

2 Morse (2015, p. 87; 2016, p. 366) claimed that in non-English sources an indicator ‘is often reported using its English name’. One wonders how he could conclude that given that he ran his searches only in English.

3 The timeframe in Morse (2015) was 1987–2012, but in Morse (2016, 2018) no start year is provided.

4 Contrarily to Factiva, TVEyes has rarely been used in academia. A search on the University of Edinburgh’s Library’s search tool for the word *TVEyes* returned only two articles in peer-reviewed journals in which this was used (Martin and McCrain, 2019; Park et al., 2020) and not even as extensively as in this article (the first one used it to cover a seven-month period, the second one only one month). A possible explanation is the higher subscription cost, as well as Factiva’s higher popularity (a basic Google search returns 6,800,000 results for the word ‘Factiva’ and only 128,000 for ‘TVEyes’).

5 Scotland’s GDP is estimated to have decreased by 21.1%, 9.5%, 6.5% and 5.4% in the second, third and fourth quarters of 2020 and first quarter of 2021 compared to the same quarters of 2019 and 2020, respectively (Scottish Government, 2020a, b, 2021a, b).

6 Data refer to the UK as a whole (i.e. all sources covered by TVEyes for the UK were included this time) because of the impossibility to filter data by source due to the huge number of references the above queries returned (which is in itself a finding).

7 Italy’s GDP decreased by 5.4%, 17.7%, 5% and 6.6% in the first, second, third and fourth quarters of 2020 compared to the same quarters of 2019, respectively (ISTAT, 2020a, b, c, 2021).

8 The MEF BES Report was actually published on February 27, but journalists seem to have picked it up one or two days later.

9 To be sure, one of the reasons is that GDP data are released monthly. However, even if one were to count the coverage of GDP as that of 12 indicators (one for each month), we must remember that the number of well-being metrics in the sample is almost twice as high, not to mention that for some well-being metrics, *too*, multiple publications are issued throughout the year (see e.g. Fig. 13).

10 Coverage of economic growth decreased in 2020 but this reflected the lack of economic growth which was balanced by an increase in interest in recession. Indeed, as soon as the economy started growing again coverage of economic growth increased and that of recession decreased.

11 To give just a flavour, there is no annual NPF Report, most indicators are not regularly updated and key publications or events related to well-being (such as the Wellbeing Economy Governments Policy Lab held in Edinburgh on May 1st, 2019) have not been press-released or promoted to the media.
